# Mitochondrial DNA somatic mutation burden and heteroplasmy are associated with chronological age, smoking, and HIV infection

**DOI:** 10.1111/acel.13018

**Published:** 2019-08-13

**Authors:** Adam S. Ziada, Meng Ying Lu, Jarek Ignas‐Menzies, Elijah Paintsil, Min Li, Onyema Ogbuagu, Sara Saberi, Anthony Y. Y. Hsieh, Beheroze Sattha, P. Richard Harrigan, Steve Kalloger, Hélène C. F. Côté, the CIHR team grant on cellular aging, HIV comorbidities in women, children (CARMA), Neora Pick, Melanie Murray, Deborah Money, Hugo Soudeyns, Fatima Kakkar, Ari Bitnun, Jason Brophy, Patricia Janssen, Joel Singer, Normand Lapointe, Jerilynn Prior, Michael Silverman, Mary Lou Smith, Heather Macdonald

**Affiliations:** ^1^ Department of Pathology and Laboratory Medicine University of British Columbia Vancouver BC Canada; ^2^ Centre for Blood Research University of British Columbia Vancouver BC Canada; ^3^ Department of Mechanical Engineering University of British Columbia Vancouver BC Canada; ^4^ Department of Pediatrics Yale School of Medicine New Haven CT USA; ^5^ School of Public Health Yale University New Haven CT USA; ^6^ Department of Pharmacology, Yale School of Medicine New Haven CT USA; ^7^ Department of Medicine Yale School of Medicine New Haven CT USA; ^8^ Department of Medicine University of British Columbia Vancouver BC Canada; ^9^ Women’s Health Research Institute Vancouver BC Canada

**Keywords:** aging, heteroplasmy, HIV, mitochondria, mtDNA, smoking, somatic mutations

## Abstract

The gradual accumulation of mitochondrial DNA (mtDNA) mutations is implicated in aging and may contribute to the accelerated aging phenotype seen with tobacco smoking and HIV infection. mtDNA mutations are thought to arise from oxidative damage; however, recent reports implicate polymerase γ errors during mtDNA replication. Investigations of somatic mtDNA mutations have been hampered by technical challenges in measuring low‐frequency mutations. We use primer ID‐based next‐generation sequencing to quantify both somatic and heteroplasmic blood mtDNA point mutations within the D‐loop, in 164 women and girls aged 2–72 years, of whom 35% were smokers and 56% were HIV‐positive. Somatic mutations and the occurrence of heteroplasmic mutations increased with age. While transitions are theorized to result from polymerase γ errors, transversions are believed to arise from DNA oxidative damage. In our study, both transition and transversion mutations were associated with age. However, transition somatic mutations were more prevalent than transversions, and no heteroplasmic transversions were observed. We also measured elevated somatic mutations, but not heteroplasmy, in association with high peak HIV viremia. Conversely, heteroplasmy was higher among smokers, but somatic mutations were not, suggesting that smoking promotes the expansion of preexisting mutations rather than de novo mutations. Taken together, our results are consistent with blood mtDNA mutations increasing with age, inferring a greater contribution of polymerase γ errors in mtDNA mutagenesis. We further suggest that smoking and HIV infection both contribute to the accumulation of mtDNA mutations, though in different ways.

## 1 INTRODUCTION

Mitochondria contain their own DNA, which code for 22 tRNAs, 2 rRNAs, and 13 proteins that are essential components of the electron transport chain. Mitochondrial DNA (mtDNA) mutations, whether inherited or acquired, have been linked to several degenerative diseases, cancers, and aging (Polyak et al., 1998; Wallace, 1999). The free radical theory of aging proposes that mtDNA, given its close physical proximity to the electron transport chain, is susceptible to oxidative damage by reactive oxygen species (ROS), leading to mtDNA damage as well as other cellular injury (Gerschman, Gilbert, Nye, Dwyer, & Fenn, 1954; Harman, 1956; Miquel, Economos, Fleming, & Johnson, 1980). Therefore, it was often assumed that ROS would increase newly acquired (somatic) mtDNA mutations, as opposed to inherited ones. However, more recent studies suggest that somatic mtDNA mutations may be predominantly introduced through replication errors by mitochondrial polymerase γ during mtDNA replication (Kennedy, Salk, Schmitt, & Loeb, 2013; Trifunovic et al., 2004). Over time, these low‐frequency mutations may undergo clonal expansion and reach the pathologic levels that contribute to aging (Payne et al., 2011). When more than one mtDNA species is observed, it is referred to as heteroplasmy. Although the association between mtDNA mutations and aging is well accepted, few studies have been able to examine the dynamics of somatic and heteroplasmic mtDNA mutations in a large cohort, in part because detecting and quantifying de novo somatic mtDNA mutations is technically challenging.

A previous study has proposed that people acquire mtDNA mutations at a rate of 6 per 10^8^ bp per year (Marcelino & Thilly, 1999). If this is true, an assay with an extremely low background error rate would be required to measure the random accumulation of such rare mutations. Currently, only primer ID (PID)‐based sequencing methods (Hiatt, Patwardhan, Turner, Lee, & Shendure, 2010; Jabara, Jones, Roach, Anderson, & Swanstrom, 2011) have achieved sufficiently low background rates (e.g., ≤10^–5^ mutations per bp) for detection of somatic mtDNA mutations. Above this threshold, detected mutations are more likely to be inherited or heteroplasmic, having arisen from clonal expansion of previously existing mutations. Given this, the definition of somatic versus heteroplasmic mtDNA mutations in the current literature may be inconsistent. Within the current study, mtDNA mutations refer to mutations present on the single strand analyzed.

People living with HIV (HIV‐positive) appear to experience premature aging (Bhatia, Ryscavage, & Taiwo, 2012). Most studies report a decrease in lifespan of ≥10 years among HIV‐positive individuals compared to HIV‐negative controls (Antiretroviral Therapy Cohort Collaboration, 2008), as well as earlier onset and higher prevalence of age‐related comorbidities (Guaraldi et al., 2011), such as cardiovascular disease (Hsue et al., 2004), some non‐AIDS‐defining cancers (Shiels, Pfeiffer, & Engels, 2010), neurocognitive decline (Ances et al., 2010), and osteoporosis (Serrano et al., 1995). Previous studies on mtDNA mutation burden in HIV have had limited sample size and focused on HIV and/or HIV therapy (Martin et al., 2003; Payne et al., 2011) without considering other potential confounders. Larger studies have either concentrated on heteroplasmic mtDNA mutations (Li et al., 2017) or used assays not sensitive enough to detect low‐frequency somatic mutations (Jitratkosol et al., 2012).

Tobacco smoking is associated with accelerated aging (Bernhard, Moser, Backovic, & Wick, 2007). Smoking increases oxidative damage, which is in turn hypothesized to cause acceleration of aging (Kiyosawa et al., 1990; Loft et al., 1992). However, no next‐generation sequencing (NGS) studies have examined the association between mtDNA mutations and smoking to date. Using other methods, increased mtDNA heteroplasmy has been linked to smoking (Lewis, Fradley, Griffiths, Baxter, & Parry, 2002; Tan et al., 2008), but evidence for mtDNA somatic point mutations is generally lacking (Coller et al., 1998). Again, these studies focused on smoking and were not powered to consider other factors that may be associated with mtDNA mutations.

The goal of the present study was to use a low background NGS assay to measure both somatic and heteroplasmic mtDNA mutation burden in relation to aging, taking into consideration important confounders of the aging process. We hypothesized that somatic mtDNA mutations increase with age, smoking, and HIV infection.

## 2 RESULTS

### 2.1 Blood somatic mtDNA substitution frequencies

A total of 92 HIV‐positive and 72 HIV‐negative Children and women: AntiRetrovirals and Markers of Aging (CARMA) participants were included in the study. Participants were all females aged 1–62 years, either current or never (but not past) smokers, and had no history of hepatitis C or B virus infection. Among all participants, chronological age was similar between the HIV‐positive (median [range] 35 [2–61]) and the HIV‐negative (32 [1–62]) groups (Table 1). The same was true for their smoking status, with 36% of HIV‐positive and 35% of HIV‐negative participants being current smokers (Table 1). However, the HIV‐positive group had lower alcohol use and a higher body mass index (BMI) (26 [23–29] vs. 22 [21–27], *p* = .01), with a different ethnic makeup (*p* < .001) from that of the HIV‐negative group. For example, 41% of the HIV‐positive individuals were African/Caribbean/Black, compared to none in the HIV‐negative group (Table 1). Among the HIV‐positive participants, 67 (73%) were currently receiving combination antiretroviral therapy (cART), 8 (9%) were cART‐naïve, and 58 (63%) had an undetectable HIV plasma viral load (pVL) at study visit.

**Table 1:** Demographic characteristics of study participants showing mtDNA substitution mutations

Parameters	All participants			Adult participants		
	HIV + (*n* = 92)	HIV − (*n* = 72)	*p*‐value	HIV + (*n* = 80)	HIV − (*n* = 59)	*p*‐value
Age (years)	35 [27–42] (2–61)	32 [24–41] (1–62)	0.134	36 [31–43] (20–61)	33 [27–43] (21–62)	0.240
BMI (kg/m^2^)				26 [23–29] (17–46)	22 [21–27] (16–53)	0.010
Smoking status						
Current smoker	33 (36)	25 (35)	1	33 (41)	25 (42)	1.000
Never smoker	59 (64)	47 (65)		47 (59)	34 (58)	
Alcohol use (drink‐year)	0 [0–7] (0–352)	2 [0–12] (0–424)	0.004	0 [0–9] (0–352)	3 [1–24] (0–424)	<0.001
Drug use (weekly/daily)						
Current or Past	26 (28)	24 (33)	0.609	26 (33)	24 (41)	0.474
Never	64 (70)	48 (67)		52 (65)	35 (59)	
Ethnicity						
Caucasian	33 (36)	32 (44)	<0.001	31 (39)	32 (54)	<0.001
African/Caribbean/Black	38 (41)	0 (0)		29 (36)	0 (0)	
Indigenous	12 (13)	14 (19)		11 (14)	14 (24)	
Asian	8 (9)	10 (14)		8 (10)	10 (17)	
Unknown	1 (1)	16 (22)		1 (1)	3 (5)	
CD4^+^ cell count (cells/μl)						
Current	510 [330–705] (30–2350)			460 [310–655] (30–1570)		
Nadir	225 [138–303] (0–2350)			210 [143–285] (0–1110)		
HIV plasma viral load (copies/ml)						
Current pVL (<50)	59 (64)			47 (59)		
Peak pVL > 100,000	40 (43)			31 (39)		
HIV treatment status						
PI‐based	46 (50)			38 (48)		
NNRTI‐based	19 (21)			16 (20)		
On other cART	2 (2)			1 (1)		
cART‐naive	8 (9)			8 (10)		
Off cART	17 (18)			17 (21)		

Note

Data presented as median [IQR] (range) or *n* (%); ethnicity is unknown for all pediatric HIV‐negative participants; cART, combination antiretroviral therapy; PI, protease inhibitor; NNRTI, non‐nucleoside reverse transcriptase inhibitor; BMI, body mass index is not presented for groups that include pediatric participants. BMI was unknown for 5 adults, current CD4+ cell count was unknown for 1 adult. pVL, HIV plasma viral load < 50 copies/ml is “undetectable.” Current pVL was unknown for 2 adults. Mann–Whitney test was used for age, alcohol use, and BMI. Chi‐square test was used for smoking status, drug use, and ethnicity.

As the somatic mtDNA substitution frequencies did not follow a normal distribution, data transformation was used and ln(*x* + 1) best normalized their distribution for linear regression modeling. All results presented hereafter refer to transformed data unless specified. Among all participants, a median [IQR] of 0.42 [0.28–0.61] transformed blood somatic mtDNA substitutions per 10,000 bp was observed. This 0.42 would translate into 0.86 mutations per mtDNA genome. Similar analyses of untransformed data are shown in Figure S1 in our Supporting Information. The assay's background error rate was calculated based on the repeated inclusion (*n* = 12) of a single cloned plasmid DNA control that functioned as a “no mutation” negative control for the assay. The assay's background somatic mtDNA substitution frequency was estimated at 0.063 [0.00–0.24] or 0.01 mutations per mtDNA genome, which was significantly lower than clinical samples (*p* < .0001; Figure S2). The assay's coefficient of variation was estimated at 6.3%, based on the inclusion of the same clinical sample in both runs (total *n* = 3).

**Figure 1:**
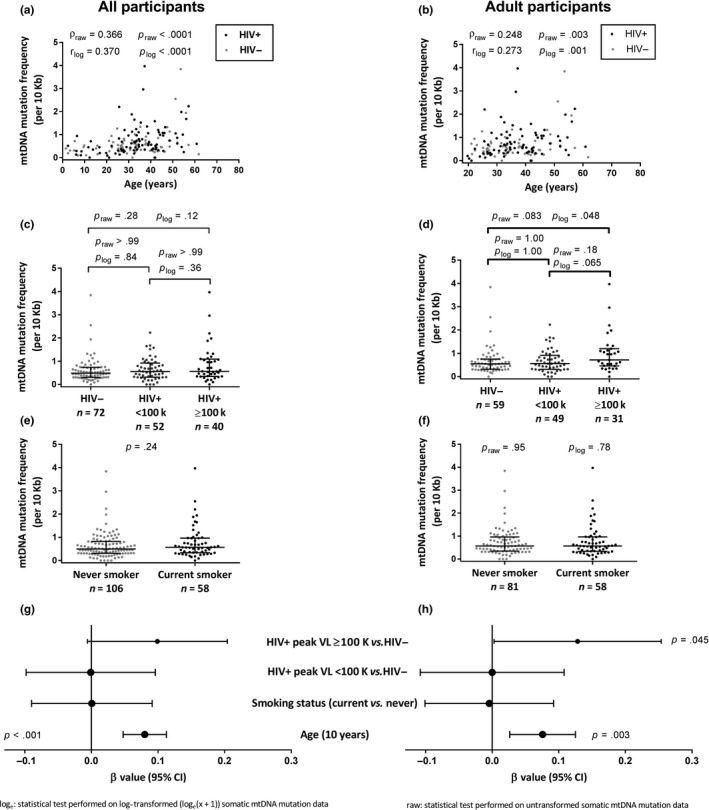
Older chronological age and high peak HIV viral load are associated with increased blood somatic mtDNA substitutions. The measured mtDNA mutation frequencies (expressed as mutations per 10,000 bp) are presented (a‐f) and p‐values for both raw and transformed (ln [x + 1]) data are shown. (a, b) Blood somatic mtDNA substitutions are positively correlated with chronological age among all participants (a) and adult participants (b). (c, d) With respect to HIV, only adult participants with a peak HIV plasma viral load ≥ 100,000 copies/ml showed a marginally higher somatic mtDNA substitution frequency compared to HIV‐negative controls. (e, f) Tobacco smoking was not associated with somatic mtDNA mutation substitutions. (g, h) Forest plot showing the estimated size of the effect (β value) and the 95% confidence interval on that estimate, based on an analysis of covariance for all (*R* ^2^ = .159) and adult (*R* ^2^ = .107) participants. The models suggest that older age remains independently associated with increased somatic mtDNA mutations, after adjusting for HIV and smoking. Furthermore, among adults, having a peak pVL ≥ 100,000 copies/ml is also associated with a higher mtDNA mutation frequency although the 95% confidence interval is wide. All univariate tests on raw data were nonparametric and used (c, d) Dunn's correction for multiple comparisons where appropriate. (c, d) ANOVA tests with Tukey's corrections. A single p‐value is reported if the same nonparametric test was used for raw and transformed data

The primary finding of our study is that these somatic mtDNA substitution mutations were significantly associated with age (*p* < .0001), whereby older individuals showed higher mutation frequencies (Figure 1). This association with age was seen among all participants (*n* = 138, *r* = .37, *p* < .001; Figure 1) and adult participants (*n* = 113, *r* = .27, *p* = .025; Figure 1).

Furthermore, transition mutations, namely A ↔ G and C ↔ T mutations, were significantly more frequent than transversion mutations (A ↔ C, G ↔ T, T ↔ A, and C ↔ G) (*p* < .0001; Figure S3b). Similar frequencies and types of mutations were seen in both HIV groups (Figure S3d). This remained true upon trichotomizing the groups according to their HIV status and peak HIV pVL (Figure S3e). The background substitutions, among the 12 cloned plasmids, also consisted primarily of transition mutations (Figure S3a).

**Figure 2:**
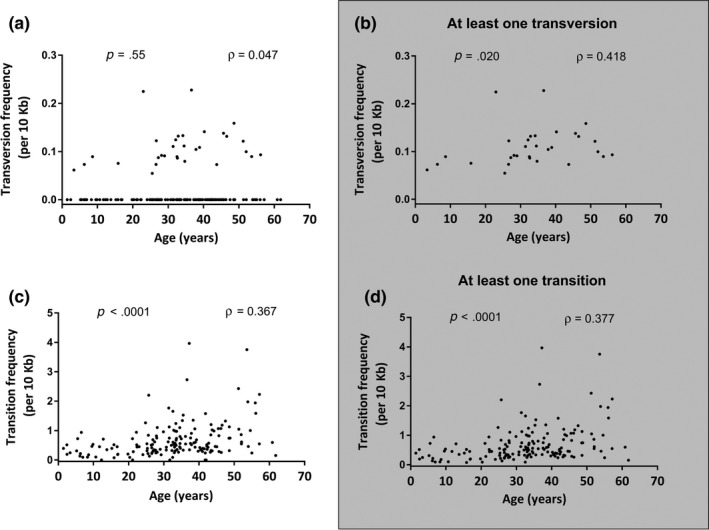
Blood mtDNA transition and transversion mutations increase with older chronological age. The measured mtDNA mutation frequencies (expressed as mutations per 10,000 bp) are presented. (a) Among all participants, no correlation between age and transversion mutation is observed; however (b) in a subanalysis removing participants with no transversion mutations, a mild correlation with increased age is seen. (c, d) Among all participants, transition mutations are associated with older age, before (c) and after (d) removing participants with no transition mutations. (a‐d) Spearman's correlations were performed on both raw and (ln[x + 1]) transformed data; a single P‐ and ρ values are presented

Among participants who had nonzero somatic mtDNA substitution frequencies (*n* = 160), plotting each type of mutation against age individually reveals that both transition (*n* = 159, *ρ* = .38, *p* < .0001) and transversion (*n* = 31, *ρ* = .42, *p* = .020) somatic mutation burden were positively associated with age (Figure 2b,d), after removing participants with a corresponding mutation (transition or transversion) frequency equal to zero, respectively.

Having established the association between somatic mtDNA substitution frequency and age, we then investigated whether HIV, a chronic infection, was associated with increased mtDNA mutation frequencies. HIV‐positive status did not show any association with somatic mtDNA substitution frequencies univariately (Table S1). However, HIV‐positive participants with a peak pVL ≥ 100,000 copies/ml had a higher somatic mtDNA substitution frequency than HIV‐negative participants (*p* = .048; Figure 1d and Table S1). Other variables investigated for possible inclusion in these models were ethnicity and BMI. Neither showed an association (*p* ≥ .1) with the somatic mtDNA substitution frequency (Table S1). Of note, there was also no evidence of an association between somatic mtDNA substitution frequency and smoking. Similarly, among HIV + adults, only HIV peak pVL and CD4 nadir showed some association (Table S1).

### 2.2 Multivariable correlates of somatic mtDNA substitution frequency

In a linear regression model of adult participants (*n* = 139, *R* ^2^ = .107) that included age, smoking status, and trichotomized HIV status, both older age and having HIV with pVL ≥ 100,000 copies/ml (vs. HIV−negative) remained independently associated with somatic mtDNA mutation burden, although the latter exhibited a large confidence interval (Figure 1h). The same model among all participants (*n* = 164, *R* ^2^ = .159; Figure 1g) only showed age as being associated with somatic mtDNA substitution frequencies. Our models describe 11% and 16% of the variance in somatic mtDNA substitutions among adult and all participants, respectively.

### 2.3 Blood mtDNA heteroplasmic substitution frequencies

**Figure 3:**
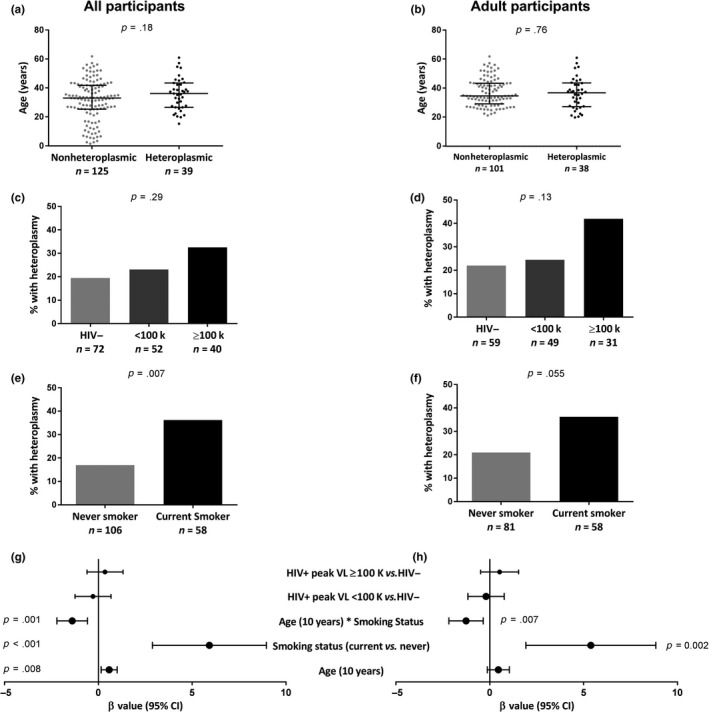
Older chronological age and tobacco smoking are associated with increased blood mtDNA heteroplasmy. Heteroplasmy was categorized as a yes/no variable. (a, b) The occurrence of heteroplasmy is not univariately associated with chronological age. (c, d) No association is observed between HIV and the occurrence of heteroplasmy. (e, f) Among all participants, current smokers show an increased occurrence of heteroplasmy (e); however, this difference is not seen among adult participants (*p* = .055) (f). (g, h) Forest plot showing the estimated size of the effect (β value) and the 95% confidence interval on that estimate, based on binary logistic regressions of all (*R* ^2^ = .339) and adult (*R* ^2^ = .308) participants. The model among all participants suggests that older age and smoking are associated with the occurrence of heteroplasmy (g); however among adults, smoking showed an association with heteroplasmy (h). Furthermore, a significant interaction between smoking and age is observed in both models

**Figure 4:**
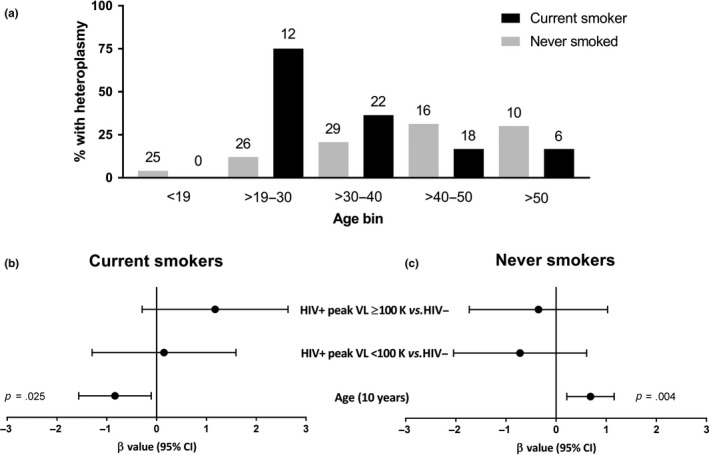
Current tobacco smokers experience a decrease in heteroplasmy with older age, while never smokers show the opposite. Heteroplasmy was categorized as a yes/no variable. (a) Percentage of study participants with heteroplasmy binned by age and smoking status, with total number of participants for each bin (above bar), and percentage of those participants (bar height). (b, c) Binary logistic regression models among current smokers (*R* ^2^ = .225) and never smokers (*R* ^2^ = .155) to further understand the effect of age on heteroplasmy. Older age is associated with a decrease in the occurrence of heteroplasmy among current smokers (b), while the reverse is seen for never smokers (c). This indicates an interaction whereby the effect of age on heteroplasmy is modulated by smoking status

Heteroplasmy was detected in 39 of the 164 participants, and information regarding heteroplasmic positions is provided in Table S2. All heteroplasmic variants were transition mutations, and the pattern of the heteroplasmic positions was different for most participants. HIV‐specific variables were also explored and again showed no association (Table S3). Only smoking status was associated with the occurrence of heteroplasmy among all (*p* = .007; Figure 3a), but not adult‐only (*p* = .055; Figure 3d) participants in univariate analysis (Table S2). An interaction was noted between age and smoking status with respect to the presence of mtDNA heteroplasmy, whereby the frequency of heteroplasmy increased with age among nonsmokers, but decreased with age among smokers (Figure 4a). Binary logistic regression models were built that included age, trichotomized HIV and peak pVL status, smoking status, and an age* smoking interaction term, for all participants (*n* = 164, *R* ^2^ = .339; Figure 3g) and adult‐only participants (*n* = 139, *R* ^2^ = .31; Figure 3h). In both models, smoking status (*p* ≤ .002) and the age*smoking interaction (*p* ≤ .007) were independently associated with heteroplasmy. Older age was also independently associated with heteroplasmy (*p* = .008) among all participants (Figure 3g). These models describe 31% and 34% of the variance in mtDNA heteroplasmy among adult and all participants, respectively. In subanalyses within smoker groups, nonsmokers showed a significant increase in heteroplasmy with increasing age (*p* = .004; Figure 4a,c), while current smokers showed the reverse, namely a decrease in heteroplasmy with age (*p* = .025, Figure 4a,b).

In an effort to independently validate this observation, data were obtained from an independent cohort at Yale New Haven Hospital (Li et al., 2017). After removing past smokers and participants with unknown smoking status, the narrow age range 53 [50–56] (30–66), and smaller sample size (*n* = 40) of the remaining participants made repeating our models within this separate cohort difficult (Figure S4). When the two cohorts were combined (results presented in Supporting Information), heteroplasmy was independently associated with older age (*p* < .001), smoking (*p* < .001), and the same age*smoking interaction term described above (Figure S5g,h) (*p* < .001), among all participants (*R* ^2^ = .24, *n* = 204; Figure S5g) and all adults (*R* ^2^ = .18, *n* = 159; Figure S5h). Further exploring the interaction term (Figure S6a), participants who currently smoked showed a nonsignificant trend toward lower heteroplasmy while they aged (*p* = .098; Figure S6b), and participants who never smoked had more heteroplasmy as they aged (*p* < .001, Figure S6c). While HIV status was univariably associated with heteroplasmy, we were unable to include it in the final multivariable models, due to the lack of peak pVL data for half of the HIV + participants in the Yale New Haven Hospital cohort.

## 3 DISCUSSION

### 3.1 Somatic mutations

We quantified somatic mtDNA substitution burden in human blood for the first time and show that somatic mtDNA substitutions increase with age. Our results therefore support the general theory that mtDNA mutations accumulate with age (Harman, 1956; Michikawa, Mazzucchelli, Bresolin, Scarlato, & Attardi, 1999). Age alone explained 14% of the variance in somatic mtDNA mutation frequency and remained the strongest independent predictor after adjusting for covariates. Our study builds on the work of a dual primer ID‐based study showing higher mtDNA somatic mutation frequencies in the brain of five elderly individuals compared to five infants (Kennedy et al., 2013). Of note, that study suggested that mutations accumulate asymmetrically on the two strands of mtDNA and that they increase with age on both strands (Kennedy et al., 2013). As our single PID method measured putative somatic mtDNA mutations on one mtDNA strand, we cannot ascertain whether these variants are present in the other strand. It is possible that some of our signal is due to chemical lesions that could be repaired or become a double‐stranded mutation. Nevertheless, our observation that these increase with age is consistent with the dual PID study (Kennedy et al., 2013).

We did not observe this age effect in our previous study restricted to mtDNA transversion (A ↔ C & T ↔ G) mutations in the blood of HIV‐positive and HIV‐negative mothers and their infants (Jitratkosol et al., 2012). This is likely related to the low sample size, the young age of the mothers, and the relative scarcity of transversion mutations seen to increase with age in our own study.

Indeed, with respect to the nature of the substitutions, transition mutations (A ↔ G, C ↔ T) were the most frequently observed, while transversion mutations (A ↔ C, A ↔ T, C ↔ G, G ↔ T) were rarely seen in our study, echoing the results seen in the above brain mtDNA study (Kennedy et al., 2013). Transition mutations are generally believed to arise from polymerase γ errors (Longley, Nguyen, Kunkel, & Copeland, 2001; Spelbrink et al., 2000), while transversion mutations are considered the signature of DNA oxidative damage. The 7,8‐dihydro‐8‐oxo‐deoxyguanosine (8‐oxodG) lesion (Yasui et al., 2014), one of the most commonly studied DNA oxidative lesions, has been shown to increase with age (Fraga, Shigenaga, Park, Degan, & Ames, 1990). However, several other lesions may also lead to transition mutations (Basu, Loechler, Leadon, & Essigmann, 1989; Kreutzer & Essigmann, 1998), and 8‐oxodG is only one of the 37 reported major products of oxidative damage to DNA (Evans, Dizdaroglu, & Cooke, 2004). Therefore, although our results do support the growing body of literature suggesting that polymerase γ errors are the major source of age‐related mtDNA mutations and variants (Kennedy et al., 2013; Trifunovic et al., 2004), we cannot rule out the potential contribution of oxidative damage as a source of mtDNA transition mutations in our study (Fraga et al., 1990; Harman, 1956; Schriner et al., 2005). In fact, among the subset of participants (19%) with detectable somatic transversion mutations, these mutations showed a weak but significant increase with age.

In addition to the effect of chronological age on somatic mtDNA substitutions, we sought to explore whether these were influenced by other factors including tobacco smoking and chronic immune activation/inflammation as seen among people living with HIV. In our sample, we failed to detect any association between somatic mtDNA substitutions and smoking. To date, no study of smoking and somatic mtDNA mutations has been conducted in blood; however, a small (*n* = 4 vs. 4) study of bronchial epithelial tissue also failed to detect any association with smoking, although the study was not sequencing‐based (Coller et al., 1998).

With respect to HIV, we only detected a significantly increased somatic mtDNA substitution burden among participants who had a high peak HIV pVL. Thus far, only two studies have investigated somatic mtDNA point mutation frequencies and HIV status. The first found significantly higher (*p* = .012) blood A ↔ C and T ↔ G transversion mutations among new mothers living with HIV (*n* = 42) than in HIV‐negative controls (*n* = 39) (Jitratkosol et al., 2012). The second investigated skeletal mtDNA and found no difference in low‐frequency mtDNA point variants (exceeding 0.2% frequency) between persons living with HIV (ART‐treated, *n* = 8, and untreated, *n* = 4) and HIV‐negative controls (*n* = 4) (Payne et al., 2011). Our study builds on these by suggesting that HIV status alone is not necessarily associated with somatic mtDNA mutation frequency, as those with a peak pVL < 100,000 copies/µl had comparable mutation frequencies to HIV‐negative individuals. Higher peak pVL may reflect poorer host control of the virus or delayed initiation of cART, both of which would increase exposure to HIV viremia and immune activation, and could potentially lead to mtDNA mutations.

Given that mtDNA mutations are estimated to accumulate at a frequency of one per 6 × 10^8^ bp per year (Marcelino & Thilly, 1999), without the application of PIDs, most sequencing‐based assays measure inherited or clonally expanded mutations rather than true somatic mtDNA mutations. Our approach exploits PIDs to distinguish sequencing errors and postprimer extension PCR errors from mutations in the original template, and is based on the prior work by other groups. These applications use either a single PID (Hiatt et al., 2010; Jabara et al., 2011) (as seen in this study) or a dual PID approach, which further eliminates possible mutations introduced during primer extension step (Schmitt et al., 2012). Since their pioneering work, PID‐based methods have been used in several fields of research including HIV (Jabara et al., 2011), cancer (Narayan et al., 2012), microbial populations (Schmitt et al., 2012), and aging (Kennedy et al., 2013). Among studies using single PIDs, reported background rates range from 1.4 × 10^−2^ to 7.6 × 10^−3^ mutations per 10,000 bp (Fox, Reid‐Bayliss, Emond, & Loeb, 2014). We measured a background error rate of 6.3 × 10^−3^ mutations per 10,000 bp, which compares favorably to the lowest single PID error rates achieved to date.

### 3.2 Heteroplasmy

The majority of studies to date have measured mtDNA heteroplasmy, defined herein as a given mutation present at >2% frequency for a given position. We report a positive relationship between older age and the presence of heteroplasmic point mutations, consistent with the literature (Michikawa et al., 1999; Munscher, Muller‐Hocker, & Kadenbach, 1993).

Furthermore, unlike somatic mutations, mtDNA heteroplasmy was significantly more prevalent among participants who were current smokers as opposed to never smokers. We also failed to detect any heteroplasmy among pediatric participants. There is an extensive body of literature linking smoking and oxidative DNA damage (Kiyosawa et al., 1990; Loft et al., 1992). Two non‐sequencing‐based studies suggest that mtDNA heteroplasmy is increased in the parotid gland (Lewis et al., 2002) and buccal cells (Tan et al., 2008) of tobacco smokers. This association of smoking with mtDNA heteroplasmy, along with the lack of association with somatic mtDNA substitutions, suggests that smoking promotes the clonal expansion of mtDNA mutations rather than the generation of de novo somatic mutations. In support of this, both increased leukocyte count (Petitti & Kipp, 1986) and mtDNA content fluctuations (Lee, Lu, Fahn, & Wei, 1998) have been associated with tobacco smoking.

The observed interaction between age and smoking with respect to mtDNA heteroplasmy, whereby the occurrence of heteroplasmy increases with age among nonsmokers but decreases among smokers has not been reported. Although our study cannot ascertain the mechanism behind this, we speculate that the higher heteroplasmic mtDNA mutation burden, or related factors in smokers, may more readily lead to the elimination of damaged cells carrying heteroplasmic mtDNA mutations. This highlights the importance of considering lifestyle confounders that may influence the frequency of detected mtDNA mutations. Unlike smoking, we did not find evidence that stressors associated with living with HIV lead to increased heteroplasmy. However, larger studies are needed to confirm this, especially since the vast majority of our participants living with HIV were treated with antiretroviral therapy and had well‐controlled HIV viremia.

Although we were unable to replicate the observed interaction between smoking and age in the Yale New Haven Hospital cohort alone, this was attributed to the narrow age range and lower sample size. When the CARMA cohort was combined with the Yale New Haven Hospital cohort, we were able to fully replicate the observations seen in the CARMA cohort alone. Large cohorts with a wide age range, information on smoking status, and next‐generation mtDNA sequencing data are very uncommon. In context, our study builds upon current knowledge on smoking and mtDNA mutation by utilizing primer ID NGS and a large cohort of individuals with a wide age range; however, further studies will be needed to confirm this interaction in an independent cohort.

To date, two small studies (Martin et al., 2003; Payne et al., 2011) and one larger study (Li et al., 2017) have investigated mtDNA heteroplasmic point mutations in persons living with HIV. The first one suggested that HIV therapy‐experienced participants showed a higher frequency of blood mtDNA heteroplasmy (*n* = 5/16) than HIV therapy‐naïve participants (*n* = 0/10). The second study demonstrated increased mtDNA deletions in muscle tissue from participants treated with HIV therapy (*n* = 21) compared to those who were therapy‐naïve (*n* = 11), but showed no evidence of increased mtDNA heteroplasmy (Payne et al., 2011). Finally Li et al. (2017) reported a significantly higher frequency of mtDNA variants among persons living with HIV and treated with cART (*n* = 47) compared to HIV‐negative controls (*n* = 24).

In our study, we found no association between HIV status and mtDNA heteroplasmy. Although the presence of heteroplasmy was somewhat more frequent among persons with a high HIV peak pVL, this did not reach significance in any model. A longitudinal study is required to confirm whether somatic mtDNA mutations, which we found more frequently among those with high peak pVL, eventually lead to heteroplasmy over time. Our study was not designed or powered to investigate the effect of cART.

### 3.3 Strengths and limitations

Our study has several strengths: (a) The use of a novel mtDNA substitution burden assay allows the quantification of very low‐frequency mutations in blood cells; (b) a large sample size facilitated multivariable modeling; (c) the broad age range of our participants, the similar behavioral characteristics between HIV groups, and the exclusion of other chronic infections (HCV and HBV) enhanced our ability to detect independent associations with age, smoking, and/or HIV.

However, the study has some limitations. As this is an observational cross‐sectional study, we cannot infer causality. Furthermore, we cannot tease apart the possible effect of HIV versus that of cART. Moreover, our analysis was restricted to mtDNA substitutions; hence, we cannot comment on mtDNA insertions and deletions. Since our analysis was restricted to the D‐loop region, we cannot generalize the observed mutation rates to the rest of the mitochondrial genome. Because we studied whole blood, we cannot address the distribution of mutations among blood cell subsets or platelets, and these could change with age, smoking, or HIV. Finally, because our study only included female participants, it may not be generalizable to males.

## 4 CONCLUSIONS

Low‐frequency mtDNA somatic substitution mutations can be quantified in blood cells, a tissue with fast turnover and lower mutation rates (Li, Schröder, Ni, Madea, & Stoneking, 2015). Our data suggest that the blood somatic mtDNA mutation burden increases with age, consistent with current thinking on biological aging, and may be increased among persons living with uncontrolled HIV. Our findings with respect to mtDNA heteroplasmy are consistent with the hypothesis that smoking promotes clonal expansion of mtDNA mutations, which may play a role in accelerating biological aging among smokers.

## 5 MATERIALS AND METHODS

### 5.1 Study sample

**Figure 5:**
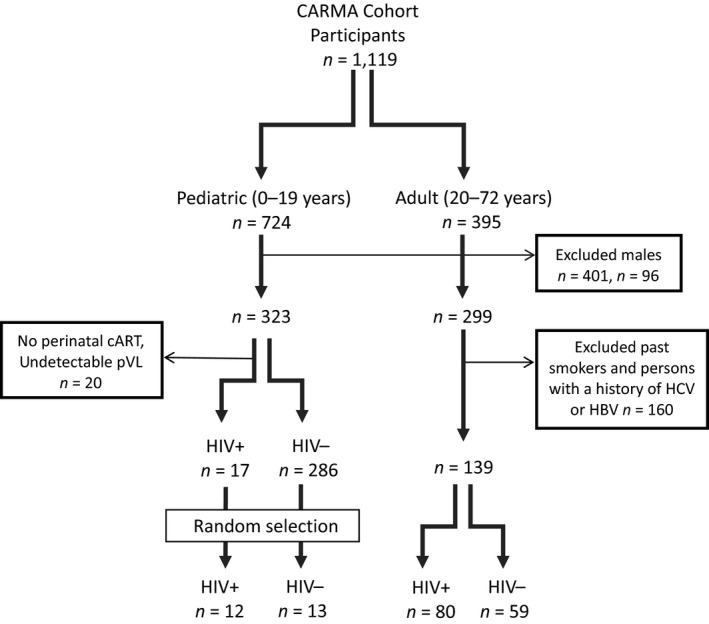
Study participants and the inclusion/exclusion criteria applied to samples analyzed. Of 1,119 CARMA cohort participants, 724 children and 395 adults were available for inclusion. As the cohort was predominantly female (75%), only females were included. Among the adults, past smokers were excluded, as were participants with a history of hepatitis C virus (HCV) or hepatitis B virus (HBV) infection, to focus on the effect of HIV. Among the 323 pediatric participants, due to restricted space in the sequencing runs, 12 HIV‐positive and 13 HIV‐negative participants were randomly selected for inclusion, all of whom were assumed HCV and HBV uninfected

Study participants were women and girls living with HIV (HIV‐positive) and HIV‐uninfected controls (HIV‐negative) enrolled in the prospective CARMA cohort. The study protocol was approved by the University of British Columbia (UBC) Clinical Research Ethics Board (H09‐02867 and H08‐02028). Each participant provided written informed consent prior to enrollment in the study. Details of the CARMA cohort study have been previously published (Cote et al., 2012; Zanet et al., 2014). Blood samples were collected between December 2008 and July 2011 in addition to relevant clinical, behavioral, and demographic information. For HIV‐negative participants under 19 years of age, leftover blood from routine testing was obtained anonymously from British Columbia (BC) Children's Hospital, and only age and sex were known for these participants (Cote et al., 2012). The inclusion criteria are presented in Figure 5.

Among all adult CARMA cohort participants, all cis‐gender females who were either current tobacco smokers or never smoked (no history of ever smoking), and with no history of infection with hepatitis B or C viruses (as per self‐report and/or serology), were included. The remaining 25 participants were selected among CARMA female pediatric participants: 12 of 37 HIV‐positive individuals who were not exposed to antiretroviral agents perinatally and 13 of 286 anonymous HIV‐negative controls. Pediatric participants were assumed to be hepatitis B‐ and C‐uninfected, and to not drink, use drugs, or smoke. In addition to HIV infection status, age, BMI, self‐reported ethnicity, drug use, alcohol use, and smoking status were also included in analyses presented here.

For HIV‐positive participants, CD4 count at time of sampling, CD4 nadir (lowest CD4 count on record), HIV plasma viral load (pVL) at time of sampling, and peak viral load (highest pVL on record) were also considered. Combination antiretroviral therapy regimen was categorized as protease inhibitor (PI)‐based, non‐nucleoside reverse transcriptase inhibitor (non‐NRTI)‐based, other class‐based, cART‐naïve, or off cART.

In this study, HIV status was trichotomized: HIV‐negative, HIV‐positive with peak pVL < 100,000 copies/ml, and HIV‐positive with peak pVL ≥ 100,000 copies/ml. This categorization was chosen based on our previous observation that having a peak HIV pVL > 100,000 copies/ml was associated with shorter leukocyte telomere length (Zanet et al., 2014), another marker of aging.

### 5.2 Somatic mtDNA substitution mutation assay

#### 5.2.1 DNA extraction and mtDNA quantification

All blood specimens were stored at −80°C until DNA extraction. Total DNA was extracted from 0.1 ml whole blood using the QIAamp DNA Mini Kit (QIAGEN, Mississauga, Ontario, Canada) on the QIAcube (QIAGEN) and eluted in 100 μl of kit AE buffer, according to the manufacturer's protocol. Each extract was then diluted 1:100 in AE (elution buffer), and mtDNA copy number was quantified by qPCR on a LightCycler 480 (Roche) with the LightCycler 480 SYBR Green I Master Kit (Roche). In brief, 2 µl of DNA was assayed in a 10 µl reaction that contained primers MT325F and MT474R (see Table S4) at a final concentration of 1 µM. The qPCR conditions were as follows: one cycle of 95°C/10 min, followed by 45 cycles of 95°C/5s; 60°C/10 s; and 72°C/5 s, with acquisition at the end of the 72°C step. Temperature ramping was 4.4°C/s for all steps except the 95 to 60°C step which ramped at 2.2°C/s. Each extract was quantified in duplicate, and the mtDNA copy numbers were determined based on a standard curve consisting of a plasmid containing the cloned amplicon, serially diluted 1:10 with a linear range extending from 3.34 × 10^7^ to 3.34 × 10^1^ copies/μl.

#### 5.2.2 Labeling with Primer IDs via single‐cycle primer extension

The 25 µl primer extension reaction contained 5 × 10^5^ copies of mtDNA, 0.5 µl of PfuUltra II fusion HS DNA polymerase and 2.5 μl of its 10× Buffer (Agilent Technologies), 0.25 mM deoxyribonucleotide triphosphate (dNTP) (Invitrogen), and 0.125 μM of a given HPLC‐purified Extension Primer (Integrated DNA Technologies) which binds the H strand of the D‐loop and extends from bp16560 of the mtDNA genome (see Table S5) according to the revised Cambridge reference sequence (https://www.mitomap.org/foswiki/bin/view/MITOMAP/HumanMitoSeq). The D‐loop region was chosen as it was less likely to be deleted compared to the rest of mitochondrial genome or have pseudogenes. A negative control designed to estimate assay background consisted of 5x10^5^ copies of a clonal plasmid DNA containing the mtDNA region of interest. Both sequencing experiments also included one or two internal controls consisting of whole blood DNA extracted from the same individual(s). Amplification conditions were 1 cycle of 95°C/140s, 60°C/20s, and 72°C/195s. The reactions were then stored at −80°C.

For every 12 participant DNA extracts assayed, three negative controls were generated by replacing the mtDNA, the extension primer, or the DNA polymerase with water. These controls were used to estimate the background incorporation of the extension primers and were taken through the subsequent steps of the assay. To prevent cross‐contamination, DNA extract labeled with the same multiplex identifier (MID) was never manipulated at the same time, and surfaces/instruments were thoroughly cleaned between handlings.

#### 5.2.3 AMPure Purification

To remove unincorporated extension primer, each reaction was combined with 50 μl of UltraPure distilled water (Invitrogen) and 50 μl of AMPure beads (Beckman Coulter) for a 10‐min incubation at room temperature, then placed into the DynaMag‐2 Magnet (Life Technologies) for 5 min, as per the manufacturer's protocol. In brief, the supernatant was removed and 200 μl of 70% ethanol was added before lightly vortexing for 5 s and placing the sample on the magnetic particle collector for 1 min. This step was repeated once more, after which the remaining supernatant was removed and the tube was placed in a 37°C heating block for 5 min. The purified product was eluted by addition of 20 μl of 10 mM Tris and 2 mM EDTA pH 7.5 for a 2‐min incubation on the magnetic particle collector. The supernatant contains the purified mtDNA, which was stored at −80°C.

#### 5.2.4 qPCR quantification of PID‐labeled mtDNA templates

The undiluted AMPure‐purified and PID‐labeled mtDNA templates were quantified via qPCR as described above using the short KSF and MT48F primers (Table S4), both at a final concentration of 1 μM. The standard curve consisted of serial 1:10 dilutions of plasmid containing the cloned amplicon, with a linear dynamic range of 1.45 × 10^7^ to 1.45 × 10^1^ copies/μl. The controls lacking DNA template, enzyme, and primer were assayed together with the respective study participant DNA extracts. The highest copy number observed among these controls (typically ≤ 10% of the participant DNA extracts) was subtracted from the copy number of the same amplified DNA sample to estimate the PID‐labeled mtDNA concentration.

#### 5.2.5 Preparation of amplicons for next‐generation sequencing

Each 50 μl PCR contained 1µl of PfuUltra II fusion HS DNA polymerase and 5 μl of its 10× Buffer (Agilent Technologies), 0.25 mM dNTP, 0.5 μM of primers LAKSF and LBDLR (Table S4), and 2,000 copies of PID‐labeled mtDNA. Amplification conditions were 1 cycle at 95°C/2 min, followed by 35 cycles of 95°C/20s, 60°C/20s, and 72°C/15s, and 1 cycle of 72°C/2 min. The PCR products were stored at −20⁰C until gel extraction.

A 2% agarose gel with a 0.5× final concentration of GelRed Nucleic Acid Gel Stain 10,000× (Biotium) was run until clear separation of the 606 bp amplicon from the lower molecular weight primer–dimer‐like material was observed. The desired band was rapidly excised under low‐intensity UV illuminator, and DNA was extracted from the gel using the QIAquick Gel Extraction Kit (QIAGEN), according to the manufacturer's protocol. Columns were wiped dry prior to elution, and incubation with elution buffer was extended to 10 min prior to the final spin. To avoid cross‐contamination within and between gel extractions, no two participants’ DNA extracts with the same MID were run in the same gel chamber, and new scalpels were used to excise each band. Between gels, running buffer was changed and all combs, gel trays, and chambers were thoroughly washed.

Each gel‐extracted product was diluted 1:40,000 in QIAGEN AE buffer and quantified by qPCR as described above, using primers MT325F and MT474R (Table S4) at 1 μM. Purified PCR products were diluted to the lowest common concentration, and 10 μl of amplicons with different PIDs was pooled. Typically, a sequencing run has approximately 10,000 reads per DNA extract. Assuming 120,000 reads per lane and 8 lanes per run, 12 amplicons were pooled in each of the 8 libraries for a total of 96 DNA samples per run.

#### 5.2.6 Single read GS FLX Sequencing

The pooled libraries were AMPure‐purified (Beckman Coulter) twice according to the manufacturer's protocol. DNA quality was then assessed via the Agilent High Sensitivity DNA Kit according to the manufacturer's instructions (Agilent Technologies). To assess the quality of the purification, each library was quantified using the KAPA Library Quantification Kit for the Roche 454 GS titanium platform as per the manufacturer's instructions (KAPA Biosystems). The libraries were then amplified following the Roche emPCR amplification method manual—Lib L SV for GS FLX + series –XL+ (May 2011)—and unidirectional sequencing was performed according to the Roche sequencing method manual for the GS FLX + instrument (May 2011). Data were processed via the default shotgun pipeline of the 454 Sequencing System Software v2.9 and output in FNA file format. All data for the present study were obtained from two full GS FLX runs, each run containing specimens from 82 study participants and 16 assay‐specific controls. The number of adults and children, HIV‐positive and HIV‐negative, and smokers/never smokers were not significantly different between the two runs. For each run, samples were selected such that the median age, and the number of smokers and participants living with HIV were similar in each run although the second run tended to have fewer children.

#### 5.2.7 Data analysis with primer IDs

FNA files were divided into 8 lanes, then split into multiple participant files based on their MID. These were subsequently sorted into groups based on their PID sequence. Consensus sequences were built for each group of sequences with identical PIDs, defined as the 19 bp immediately following the MID. These were then used to build a consensus sequence for each participant, based on all sequences sharing the same MID in a given lane. Sequences that did not reach position 279 in the mtDNA genome or did not contain one of the 12 MIDs used (Table S5) within the first 100 bp were removed from the analysis.

Mafft sequence aligner within Unipro UGENE v1.24.1 (http://ugene.net/) was used, with the opening penalty set at 0.05, the extension penalty at 0.05, and the max iterations at 10. PID consensus sequences were built from ≥5 distinct sequences or reads. Each participant's consensus sequence was built from ≥100 PID consensus sequences. A mutation was defined as any difference between the participant DNA extract's consensus sequence and the PID’s consensus sequence, if the mutation was present in ≥75% of the ≥5 sequences used to build the PID consensus sequence. These thresholds were established a priori, based on pilot work. Only substitution mutations between bp 16,560 and 279 (see Figure S7) of the mitochondrial D‐loop, based on the revised Cambridge reference sequence, were analyzed. Somatic mtDNA substitution mutations were defined as mutations occurring in ≤2% of PID consensus sequences for a given participant. Mutations detected in the same position in >2% of PID consensus sequences were defined as heteroplasmic. Within the current study, mtDNA mutations refer to mutations present on the single strand analyzed. Insertions and deletions were not considered in this analysis. Both code and data used in this manuscript are available upon request.

Our main outcome of interest was somatic mtDNA substitution mutation burden, reported as substitutions per 10,000 bp, extrapolated from the 289‐bp interrogated region in the mitochondrial D‐loop. Only PID groups with ≥5 sequences were considered for analysis. As such, the somatic mtDNA substitution frequency = (number of somatic mtDNA substitutions × 10,000) divided by (number of distinct PID groups with 5 or more sequences × 289 bp).

**Figure 6:**
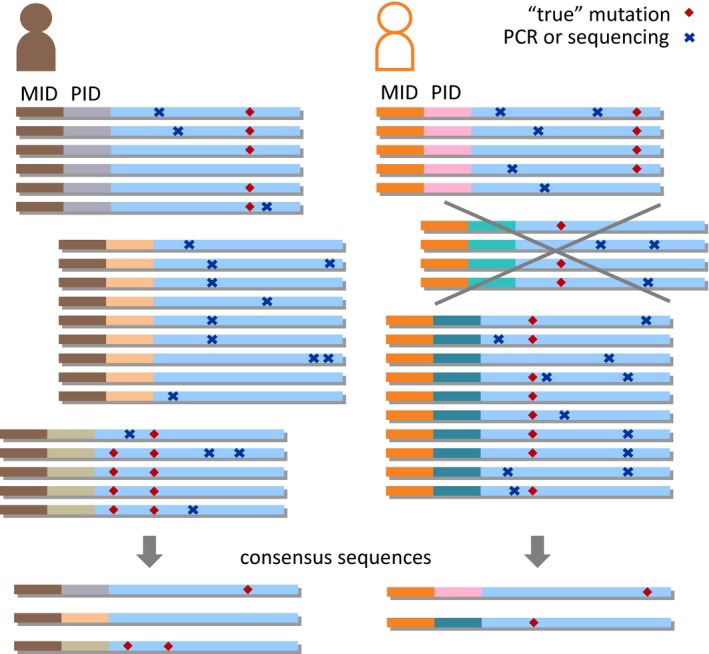
Schematic of the mtDNA mutation assay. Sequences belonging to a given individual are grouped according to their multiplex identifier (MID) and according to their mtDNA primer identifier (PID). A consensus sequence is generated for each individual, based on all sequence groups containing at least five reads. Each group’s consensus sequence is then compared to the individuals’ consensus sequence. If a mutation (i.e., not present in the individual’ consensus sequence) is present in ≥75% of all reads belonging to a given group, it is categorized as a “true” mutation which is presumed to have been present in the original mtDNA molecule, at the time of primer labeling. All other mutations are deemed to represent PCR errors that occurred after the initial single‐cycle primer extension step or sequencing errors

mtDNA heteroplasmy was reported as a binary variable (yes/no). Figure 6 summarizes the principle of the assay, which exploits the fact that true mutations present in the original mtDNA template will be present in a majority of sequences sharing the same PID (established as ≥75% here). Mutations caused by PCR errors after the single‐cycle primer extension step or sequencing errors will result in a scattered pattern within PID groups.

### 5.3 Validation in an independent cohort

Furthermore, we validated our results using data from an independent cohort enrolled at Yale New Haven Hospital, New Haven, Connecticut, USA (Li et al., 2017) (Li et al., 2017). We included in the current analysis data from study participants living with HIV who were either current smokers or never smokers. Although the original study included HIV‐negative controls, smoking status was not available for those individuals. Only substitution mutations within the same 289‐bp region of the mitochondrial D‐loop were considered.

### 5.4 Statistics for somatic mtDNA substitutions

Given the non‐normal distribution of the raw data, somatic mtDNA substitution frequencies were ln(*x* + 1)‐transformed, where *x* is the somatic mtDNA substitution frequency per 10,000 bp. Comparisons between the HIV‐positive and HIV‐negative groups were done using the Mann–Whitney U and Fisher's exact tests as appropriate. Univariate associations between (transformed) somatic mtDNA substitution mutation burdens and possible explanatory variables were investigated with linear regressions (age, log‐transformed BMI, current CD4 count), Spearman's correlation (CD4 nadir), two‐sample t or Mann–Whitney U tests (smoking status, current HIV pVL, peak pVL), one‐way ANOVA, or Kruskal–Wallis (trichotomized HIV status, HIV treatment status, and ethnicity), as appropriate. An a priori decision was made to include age, smoking status, and HIV status in all models. Other variables were included in the ANCOVA multivariable model if they were important univariately (*p* < .1), and improved the fit of the multivariable model.

### 5.5 Statistics for heteroplasmic mtDNA substitutions

Mann–Whitney *U* or Fisher's exact tests were used to compare groups and assess univariate associations in the presence or absence of heteroplasmy. For the multivariable modes, variables were selected as above for inclusion in the binary logistic regression model.

### 5.6 Statistics used to analyze specific mtDNA substitution types

Mann–Whitney *U* tests or Kruskal–Wallis tests with a Dunn's correction for multiple comparisons were used to compare the somatic mutation frequency of specific types of mutations. Spearman's correlation was used to assess their relationship with age.

### 5.7 Statistical software

Statistical analyses were performed with XLSTAT Version 2013.1.01 (Addinsoft) and JMP software, v. 12.2.0 (SAS Institute), with the exception of Dunn's and Tukey's corrections, which were done with GraphPad Prism version 7.01 (GraphPad Software).

## CONFLICT OF INTEREST

None declared.

## AUTHOR CONTRIBUTIONS

A.S.Z., MY.L., S.S., P.R.H., and H.C.F.C. designed research; A.S.Z., M.L., O.O., and B.S. performed research; A.S.Z. and J.IM. contributed new reagents/analytic tools; A.S.Z., S.K., and H.C.F.C. analyzed data; A.S.Z., E.P., A.Y.Y.H., and H.C.F.C wrote the paper; A.S.Z., MY.L., S.S., P.R.H., and H.C.F.C designed the NGS assay; E.P. designed the validation study; M.L. and O.O. revised the manuscript.

## APPENDIX A

Other investigators of the CIHR Team in Cellular Aging and HIV Comorbidities in Women and Children (CARMA) include: Neora Pick, MD; Melanie Murray, MD; Deborah Money, MD; Hugo Soudeyns, PhD; Fatima Kakkar, MD; Ari Bitnun, MD; Jason Brophy, MD; Patricia Janssen, PhD; Joel Singer, PhD; Normand Lapointe, MD; Jerilynn Prior, MD; Michael Silverman, MD; Mary Lou Smith, PhD; Heather Macdonald, PhD.
