# Supplementary figures and images for: Mitochondrial DNA somatic mutation burden and heteroplasmy are associated with chronological age, smoking, and HIV infection
Source: Aging Cell. 2019 Aug 13;18(6):e13018. doi: 10.1111/acel.13018 (PMC6826146; doi:10.1111/acel.13018)

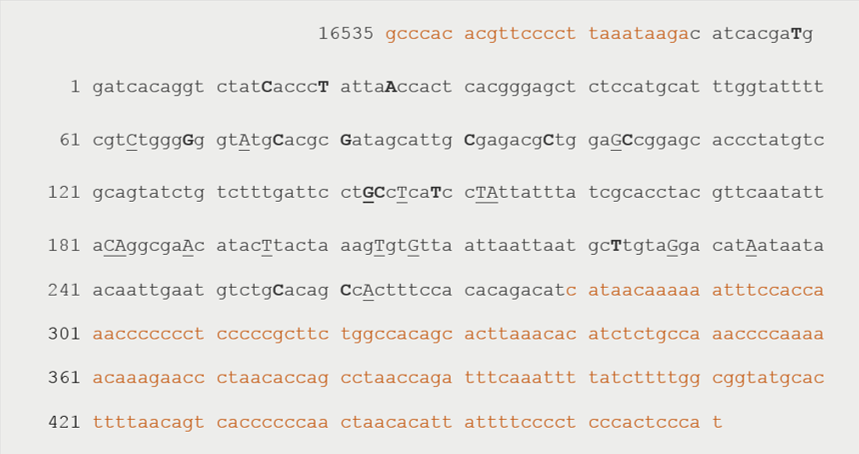

Supplement: Supplementary file 1 [file ACEL-18-e13018-s001.png]

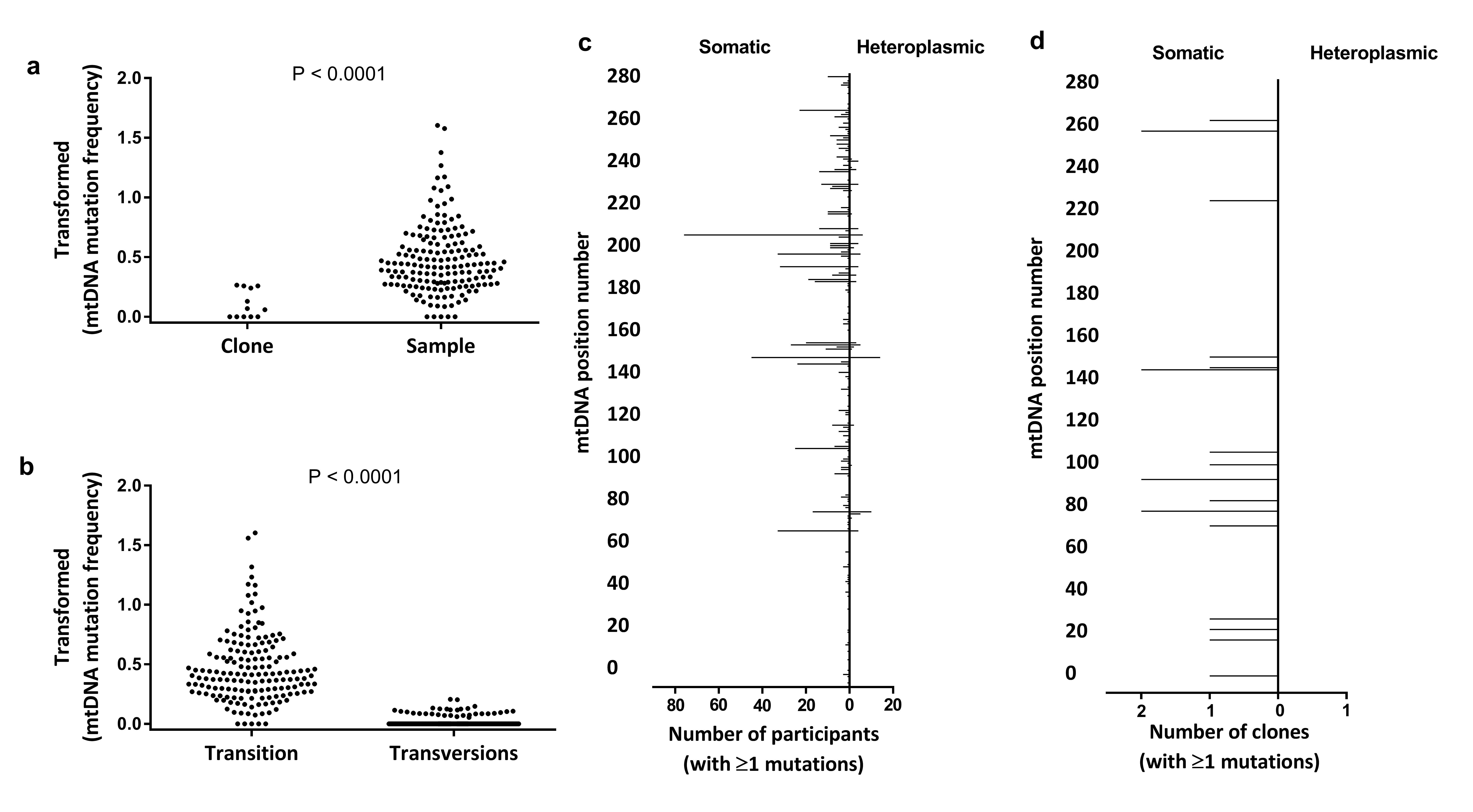

Supplement: Supplementary file 2 [file ACEL-18-e13018-s002.jpg]

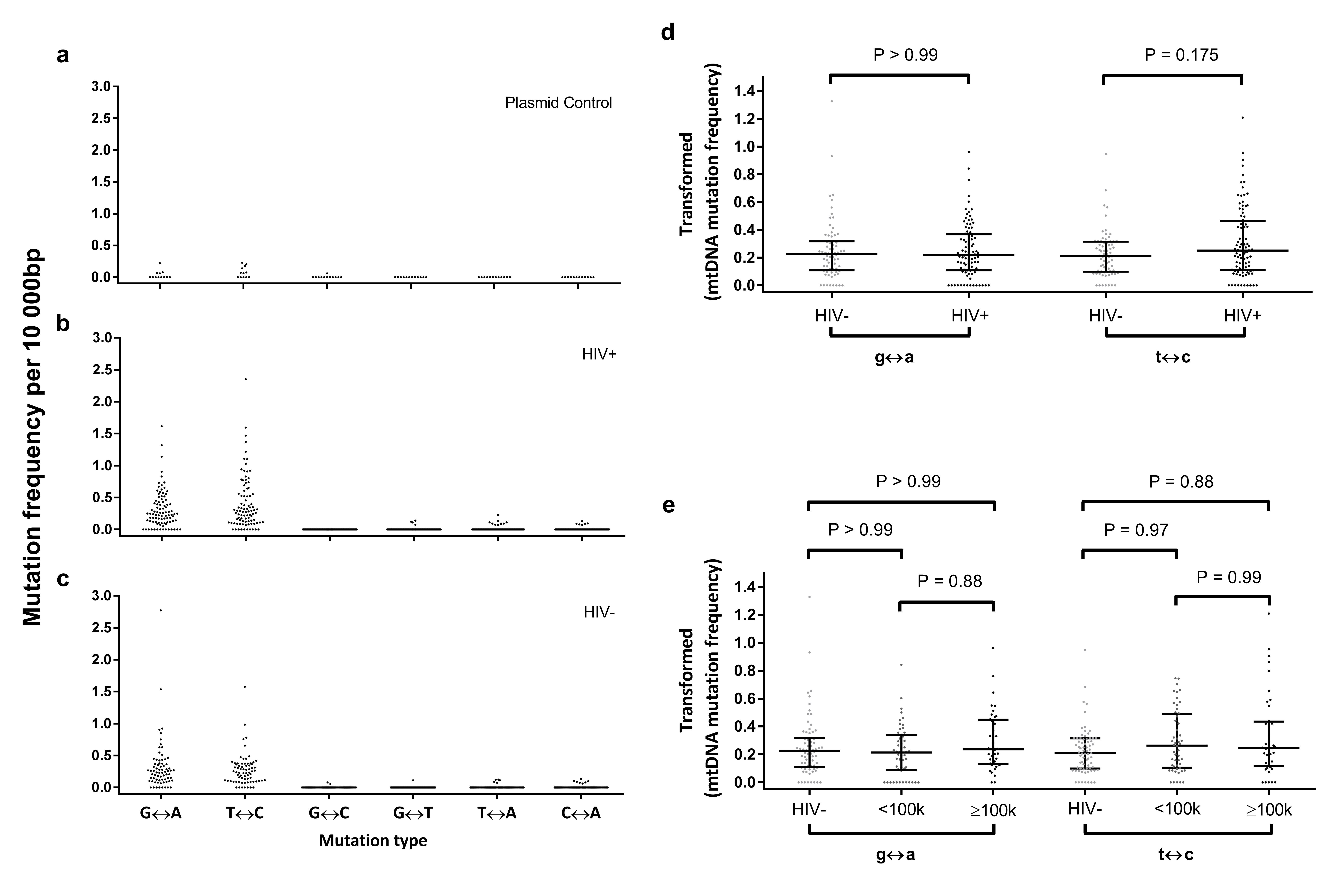

Supplement: Supplementary file 3 [file ACEL-18-e13018-s003.jpg]

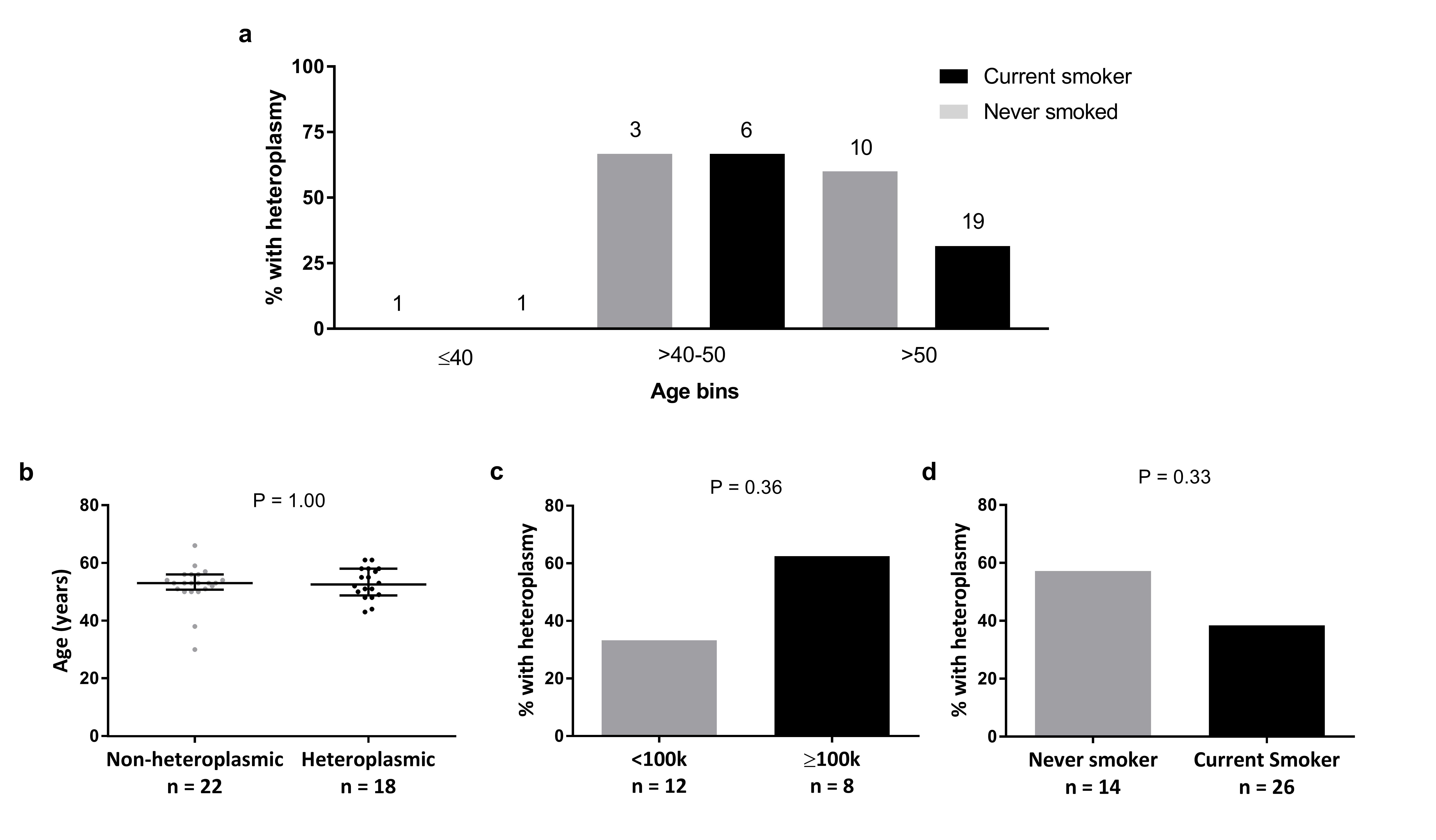

Supplement: Supplementary file 4 [file ACEL-18-e13018-s004.jpg]

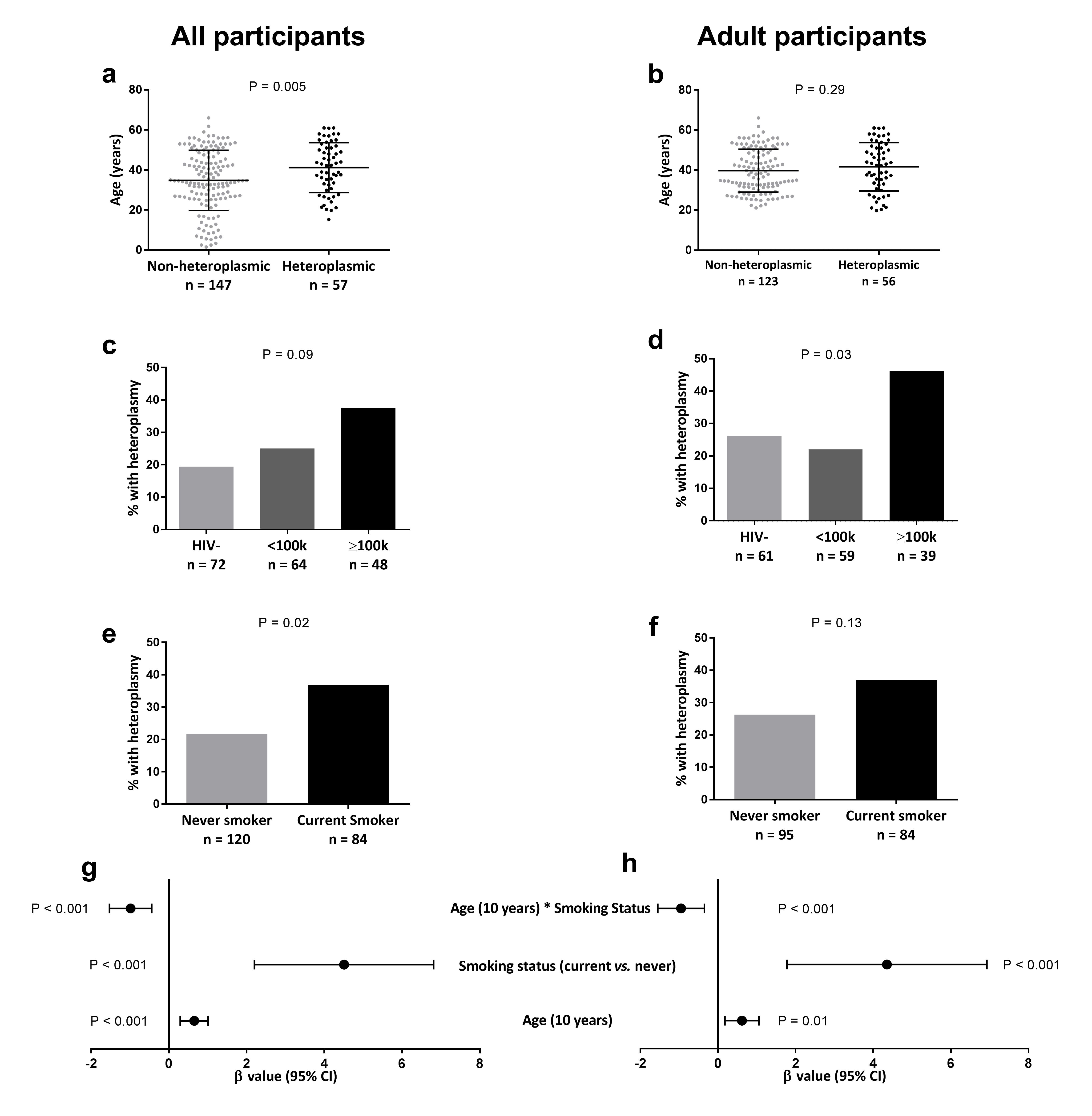

Supplement: Supplementary file 5 [file ACEL-18-e13018-s005.jpg]

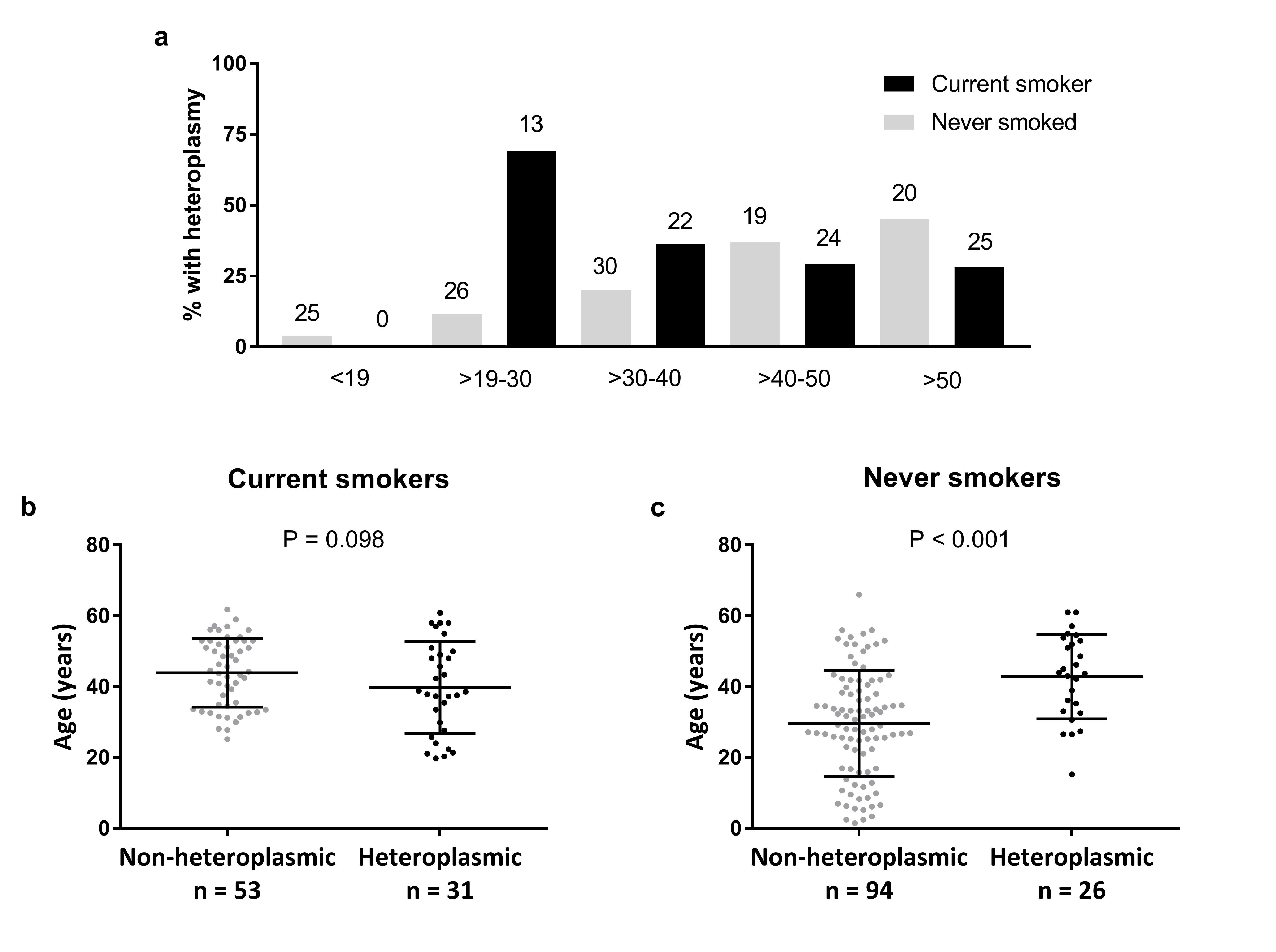

Supplement: Supplementary file 6 [file ACEL-18-e13018-s006.jpg]

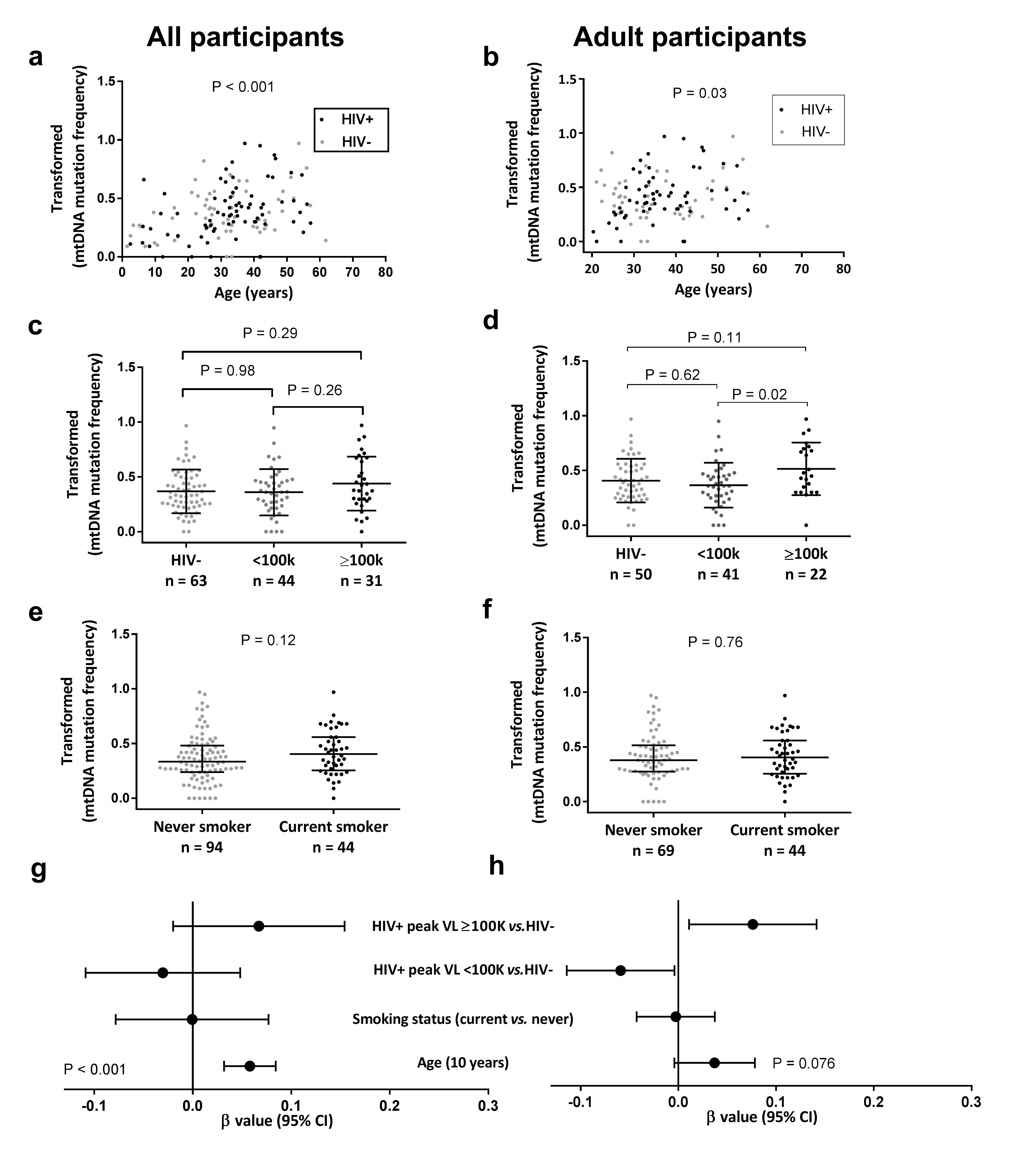

Supplement: Supplementary file 7 [file ACEL-18-e13018-s007.jpg]

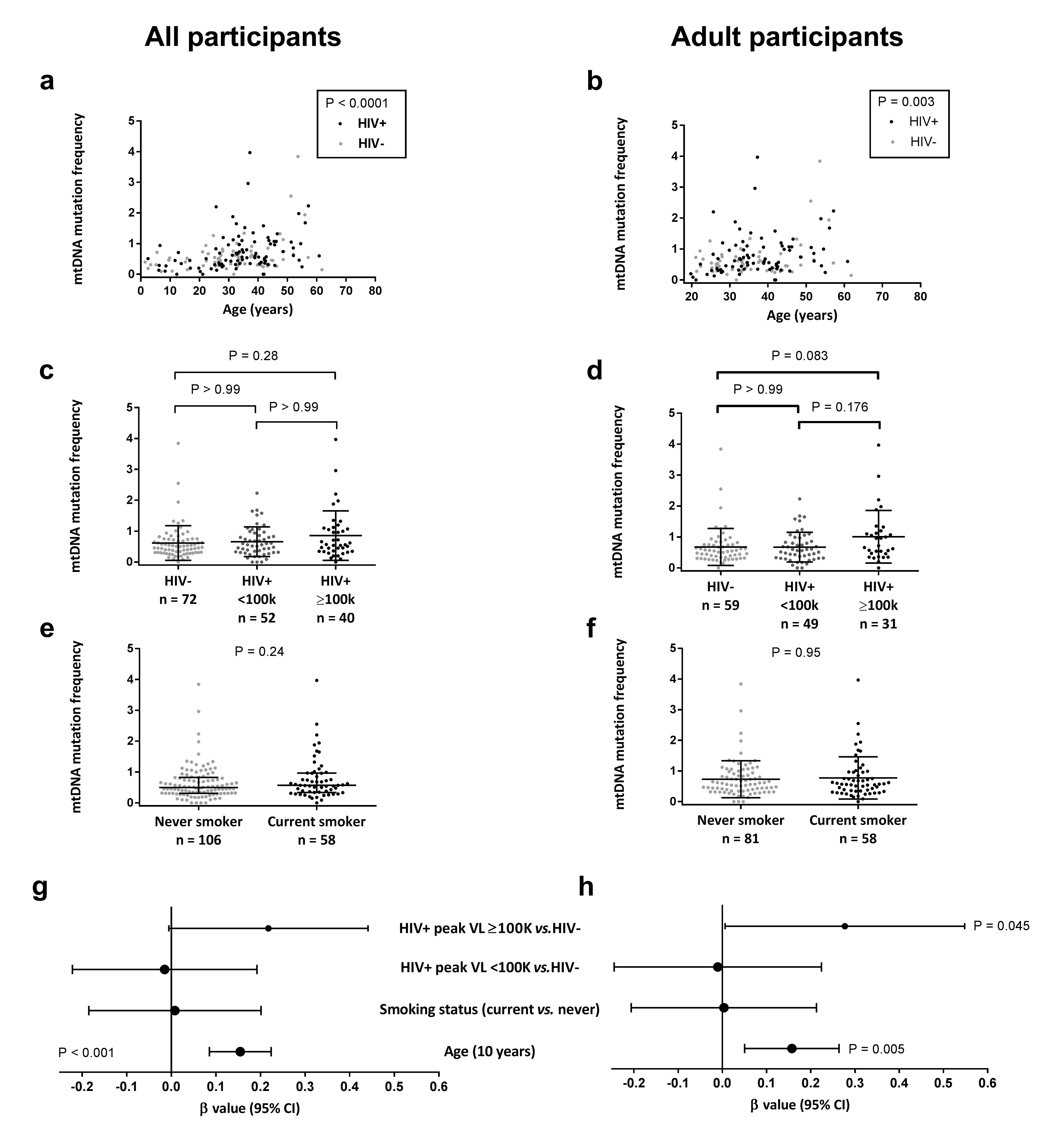

Supplement: Supplementary file 8 [file ACEL-18-e13018-s008.jpg]
