# Supplementary material for: Mitochondrial DNA somatic mutation burden and heteroplasmy are associated with chronological age, smoking, and HIV infection
Source: Aging Cell. 2019 Aug 13;18(6):e13018. doi: 10.1111/acel.13018 (PMC6826146; doi:10.1111/acel.13018)
Supplement: Supplementary file 10 [file ACEL-18-e13018-s010.doc]

Supporting Information for

Mitochondrial DNA somatic mutation burden and heteroplasmy are associated with chronological age, smoking, and HIV infection

Adam Sherif Ziada, Meng Ying Lu, Jarek Ignas-Menzies, Elijah Paintsil, Min Li, Onyema Ogbuagu, Sara Saberi, Anthony Y. Y. Hsieh, Beheroze Sattha, P. Richard Harrigan, Steve Kalloger, Hélène C.F. Côté and the CIHR team grant on cellular aging and HIV comorbidities in women and children (CARMA)*.

*Other investigators of the CIHR Team in Cellular Aging and HIV Comorbidities in Women and Children (CARMA) include: Neora Pick, MD; Melanie Murray, MD; Deborah Money, MD; Hugo Soudeyns, PhD; Fatima Kakkar, MD; Ari Bitnun, MD; Jason Brophy, MD; Patricia Janssen, PhD; Joel Singer, PhD; Normand Lapointe, MD; Jerilynn Prior, MD; Michael Silverman, MD; Mary Lou Smith, PhD; Heather Macdonald, PhD.

Hélène Côté

Email: helene.cote@ubc.ca

**This PDF file includes:**

Supplementary text

Figures S1 to S8

Tables S1 to S10

References for SI reference citations

**Short reads and reads without a PID and/or MID**

Sequencing data was obtained for all 164 participants and 12 plasmid controls. Approximately 11/11791 (0.09%) of reads were removed because their length did not meet quality control (QC) parameters, while 93/11791 (0.79%) were removed for failure to contain an expected MID or the PID.

**Drug and alcohol use**

The drug use (weekly/daily) current or past refers to participants self-reporting using illicit drugs (including cannabis) on a daily to weekly basis during their life, excluding those who only experimented with or used drugs infrequently in their lifetime (Zanet et al., 2014). For alcohol consumption, the drink-year variable (drinks/day*years drinking) was calculated by multiplying the number of drinks per day by the number of years the participants self-reported consuming alcohol.

Among all participants, alcohol (P = 0.005) but not drug (P =0.084) use were univariately associated with somatic mtDNA substitution frequency. This was not true among adult participants, were neither drug (P = 0.447) nor alcohol (P = 0.431) were associated with somatic mtDNA substitution frequency (Table S3). While both alcohol and drug use qualified (P < 0.01) for inclusion in our multivariable models among all participants, both failed to improve the model after correcting for age and so were not in the final model.

Univariately, heteroplasmy tended towards an association with alcohol use among all participants (P = 0.072), but not adult participants (P = 0.503). Drug use showed no univariate associations with heteroplasmy (Table S5). While alcohol use would have qualified for inclusion (p < 0.1) in our multivariable model of heteroplasmy among all participants, its inclusion failed to substantially improve the model and so it was subsequently removed from the model.

Overall, the univariate associations seen for drug use and alcohol were likely related to the inclusion of pediatric participants who do not drink or use drugs. This is further supported by the fact that these associations were not observed among adults and showed no independebnt association after adjusting for age.

**Mutations and mtDNA D-loop position hotspots**

Of 289 D-loop positions examined in this analysis, 159 showed a somatic mutation, and 27 were heteroplasmic in at least one participant (Figure S2c). The vast majority (23/27) of positions displaying heteroplasmy were also locations where somatic mutations were observed, typically the exact same mutation (22/23) (Figure S2c). No heteroplasmy was observed in any of the 12 plasmid clones. However somatic mutations were observed at 16 positions. Of these, none corresponded to heteroplasmic positions, and 9 were the site of somatic mutations among participants (Figure S2c,d).

**Sensitivity analysis with high stringency somatic mutation filter**

We conducted these experiments with the heightened awareness that contamination between participants would inflate mutation frequencies, presented in the supplement. As part of our QC, and in order to be certain the results were not a consequence of such cross-participant contamination, we sought to apply an extremely stringent filter to our data. We then determined whether this affected our primary findings with respect to somatic substitutions, knowing that this filter would de facto result in a severe underestimation of heteroplasmy. In brief, each PID consensus sequences containing ≥1 substitution mutations was compared to the consensus sequences of each DNA extract analyzed within the same 454 GS FLX run. Extracts containing more than 20 PID consensus sequences perfectly matching the consensus sequence of another extract in the same run—excluding insertions and deletions—were removed from the analysis. Among the remaining extracts, PID consensus sequences containing ≥2 substitution mutations were compared to the consensus sequences of every participant and control within the same GS FLX run. Any PID consensus sequences containing ≥2 substitution mutations and perfectly matching the consensus sequence of another extract in the same run —excluding insertions and deletions— were identified and removed. Given the low likelihood of ≥2 distinct mutations occurring within the same 289-bp DNA sequence at the same position, these were treated as potential contaminations, hence removed from this sensitivity analysis. The minimum number of distinct PID consensus sequences per extract was also raised to 200 for inclusion in this analysis.

Statistics were done similarly to those presented in the main paper, with the exception that Least Square linear regression was used for the adult multi-variable model instead of an ANCOVA.

Applying the high stringency somatic mtDNA mutation filter described in the method section, 26 participants were removed from the analysis (24 for having >20 PID in common with others in the run, 2 for having <200 PID consensus sequences). Among the remaining 138 participants, 41 had at least one PID consensus sequence removed. Age and smoking status remained well balanced between the HIV-positive (n = 75) and HIV-negative (n = 65) participants while BMI and ethnicity were not (Table S6).

As seen with the original dataset, somatic mtDNA substitution frequencies were univariately associated with older age, among all (P < 0.0001), all adult (P = 0.004), and HIV-positive adult (P = 0.007) participants (Figure S8). A peak HIV pVL ≥100 000 copies/mL also remained associated among all adults and HIV-positive adults (Table S7). Although alcohol (P < 0.01) and drug (P = 0.03) use were significantly associated with somatic mtDNA substitution frequencies among adults (Table S7), their inclusion failed to substantially improve the multivariable model (data not shown). In an analysis of covariance model of all participants, the results were similar to those obtained with the original dataset (Figure S8g). In a least squares regression model of all adults, a higher somatic mtDNA substitution frequency was independently associated with having a peak pVL ≥100 000 copies/mL (vs. HIV-negative) while the reverse was seen for those with a peak pVL <100 000 copies/mL (vs. HIV-negative), Figure S8h. Of note, in that model, age was no longer significantly associated with somatic mtDNA substitution frequency (P = 0.076).

As might be expected, applying this high stringency somatic mtDNA mutation filter removed 24/39 (62%) samples containing heteroplasmy. Additionally, among the remaining 15, the heteroplasmy of 5 (13%) participants was decreased below the threshold (<2%) to be categorized as heteroplasmy, leaving too few (n = 10) to perform statistics.

When dealing with extremely rare somatic mutations, even low levels of DNA contamination can be problematic. However, applying an extremely stringent filter to our data in order to remove possible cross-sample or cross-run contamination (Brumme et al., 2017) yielded similar results, reinforcing the notion that such contamination, should it be present, did not affect our findings.


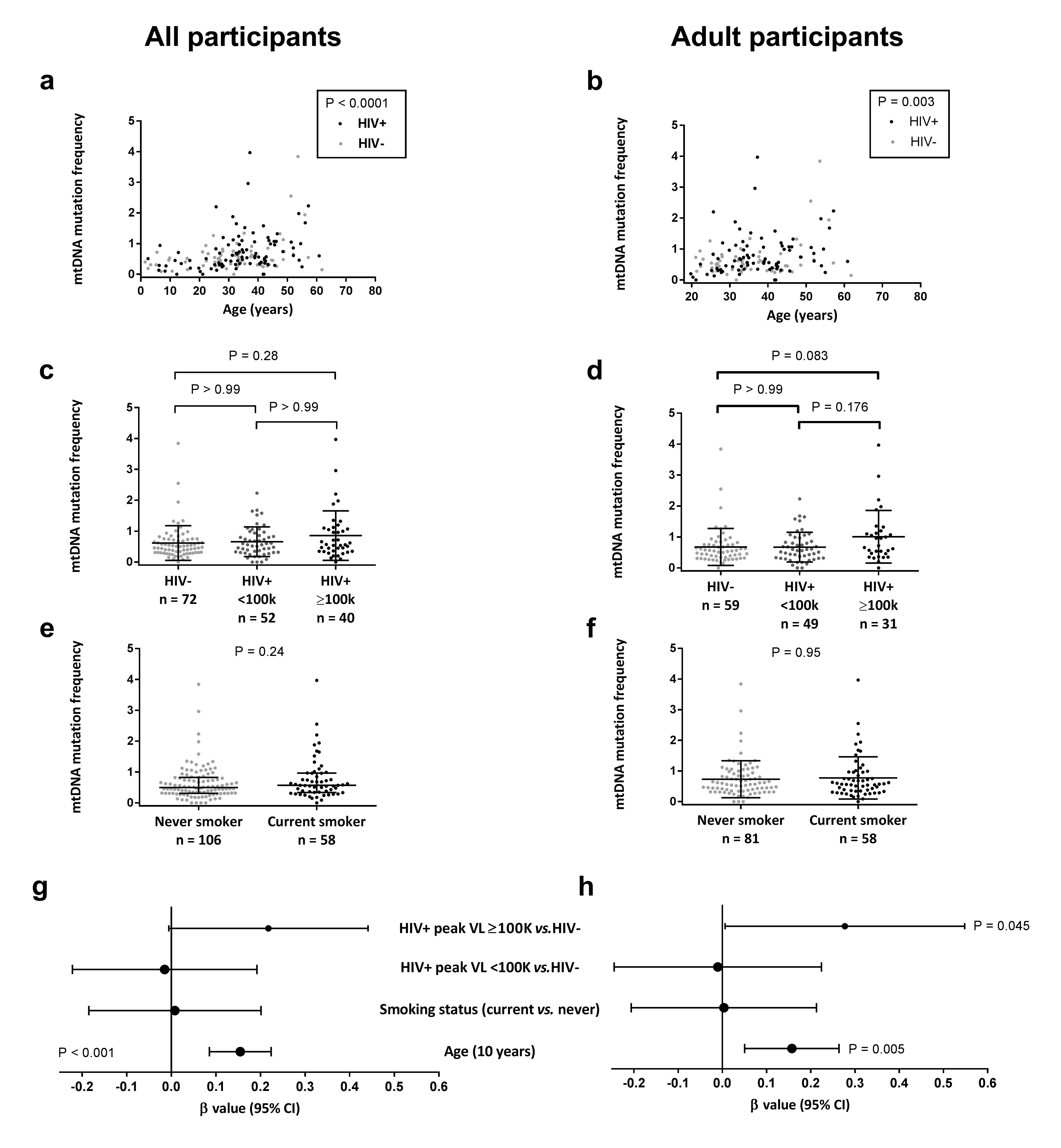


**Figure S1** Older chronological age and high peak HIV viral load are associated with increased blood somatic mtDNA substitutions. The measured mtDNA mutation frequencies (expressed as mutations per 10,000 bp) in untransformed data. (**a**, **b**) Blood somatic mtDNA substitutions are positively correlated with chronological age among all participants (**a**) and adult participants (**b**). (**c**, **d**) With respect to HIV, only adult participants with a peak HIV plasma viral load ≥100,000 copies/mL tended to have a marginally but non-significantly higher somatic mtDNA substitution frequency compared to HIV-negative controls (P = 0.083) or HIV-positive participants with a peak VL <100,000 copies/mL (P = 0.176) (**d**). (**e**, **f**) Tobacco smoking was not associated with somatic mtDNA mutation substitutions. (**g**, **h**) Forest plot showing the estimated size of the effect (β value) and the 95% confidence interval on that estimate, based on an analysis of covariance. The models suggest that older age remains independently associated with increased somatic mtDNA mutations, after adjusting for HIV and smoking. Furthermore, among adults, having a peak VL ≥100,000 copies/mL is also associated with a higher mtDNA mutation frequency although the 95% confidence interval is wide. Spearman correlations, Kruskal-Wallis tests with a Dunn’s correction for multiple comparisons, and Mann-Whitney U tests were used in (**a**), (**b**) (**c**), (**d**), (**e**) and (**f**) as appropriate.


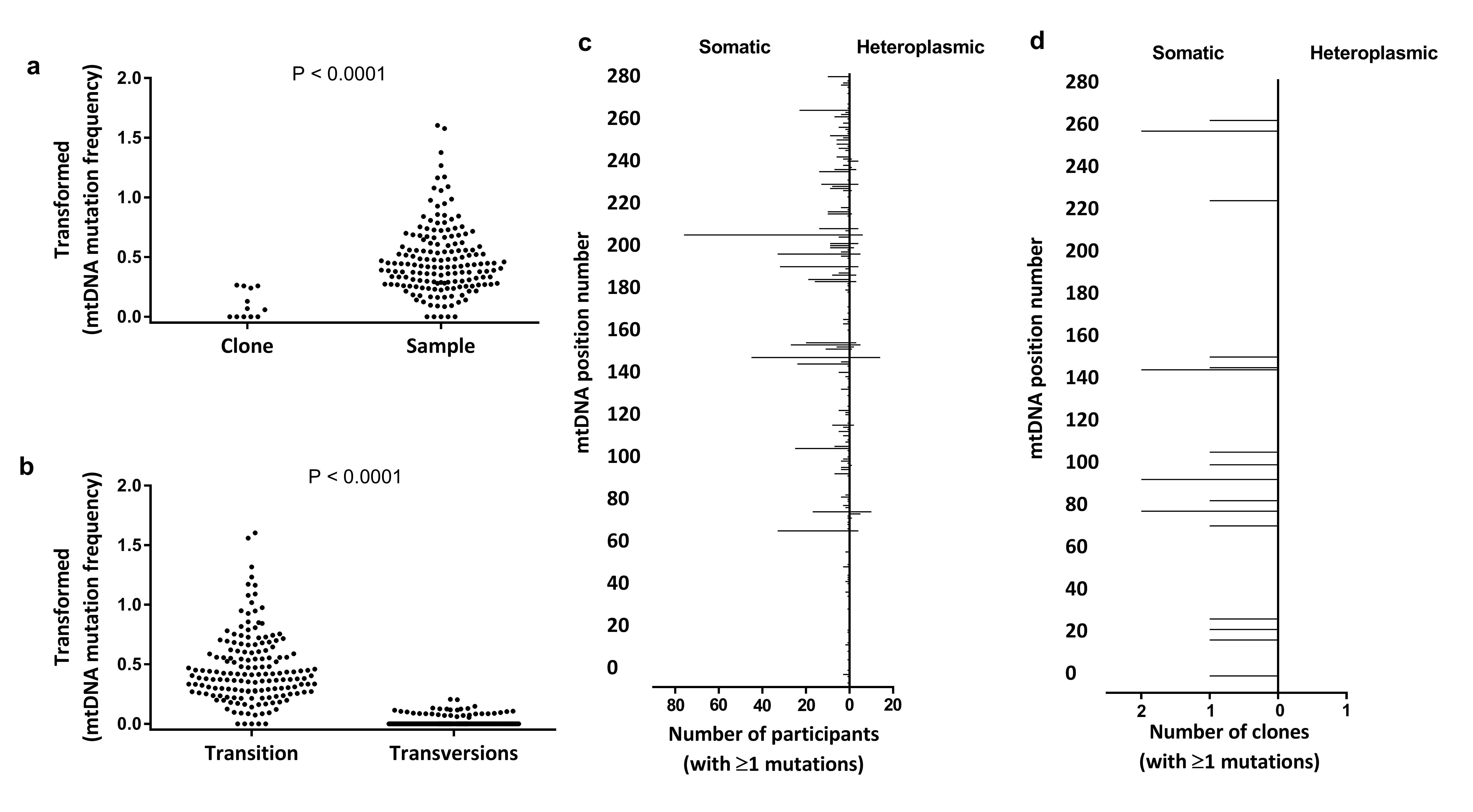


**Figure S2** Low background mutation rate and predominance of transition mtDNA mutations. (**a**) The blood somatic mtDNA substitution mutation frequency was measured 12 independent times for a cloned plasmid, and once for 164 distinct participants. The frequencies (expressed as mutations per 10,000 bp) were transformed (ln [x+1]) to normalize the distribution. The assay background error rate (cloned plasmid) is significantly lower than that observed for study participants (blood) (p < 0.0001). (**b**) Among the 164 participants, both mtDNA transition and transversion mutations are observed, however the vast majority are transition mutations. (**c**) Depiction of the number participants harbouring one or more somatic mtDNA substitutions (left of the Y axis), or heteroplasmy (right of the Y axis) at a given mtDNA position (mtDNA 16560 [or here -10] to 279) of the D-loop accoding to the revised Cambridge reference sequence. MtDNA heteroplasmy and somatic substitutions are seen throughout the region sequenced. Higher mutation frequencies occur at positions 64, 146, and 204. (**d**) Illustration of the number of cloned plasmids harbouring one or more somatic mtDNA substitutions or heteroplasmy at a given position. Somatic mutations are present in the clones at 16 distinct positions. Among participants, no heteroplasmy is observed at any of these 16 positions but somatic mutations are seen at 9 of these. Mann-Whitney U tests were used in (a) and (b).


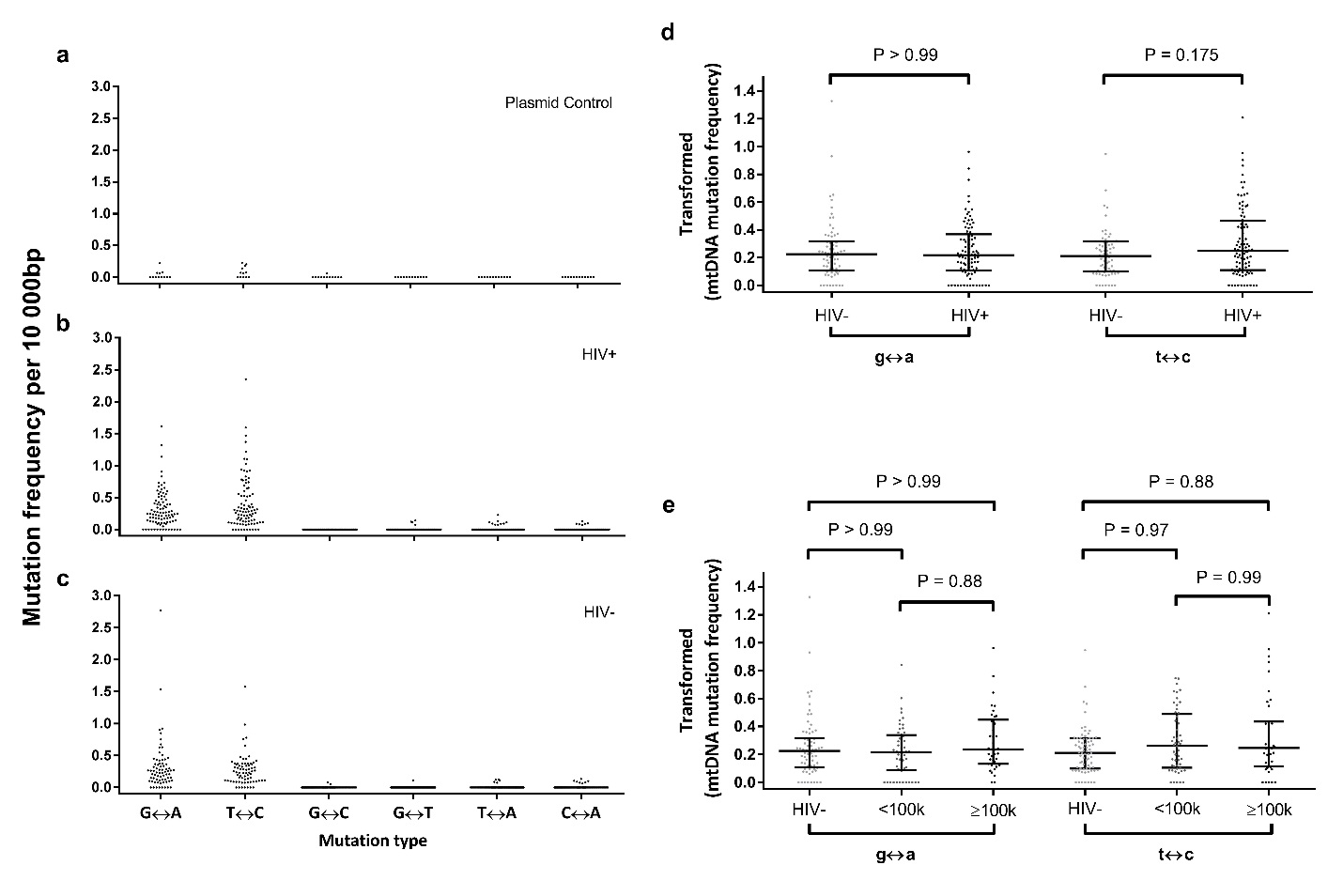


**Figure S3** No observable difference in the mutation signature of HIV positive and HIV-negative participants. For all 92 HIV-positive (**b**), and 72 HIV-negative (**c**) study participants and 12 plasmid clonal controls (**a**), each sample’s mutation frequency was calculated for different types of transversion and transition mutations. The measured frequencies (expressed as mutations per 10,000 bp) were transformed (ln [x+1]) to normalize the distribution. (**d**) A sub analysis of G↔A and T↔C transition mutations, shows no significant difference in either type of mutation between HIV-positive and HIV-negative participants. (**e**) Furthermore, when HIV status is trichotomized to include both HIV-positive individuals with a peak HIV pVL ≥ and <100 000 copies/mL, no relationship is observed between G↔A or T↔C transition mutations and trichotomized HIV status. Kruskal-Wallis tests with a Dunn’s correction for multiple comparisons (**d**) and (**e**).


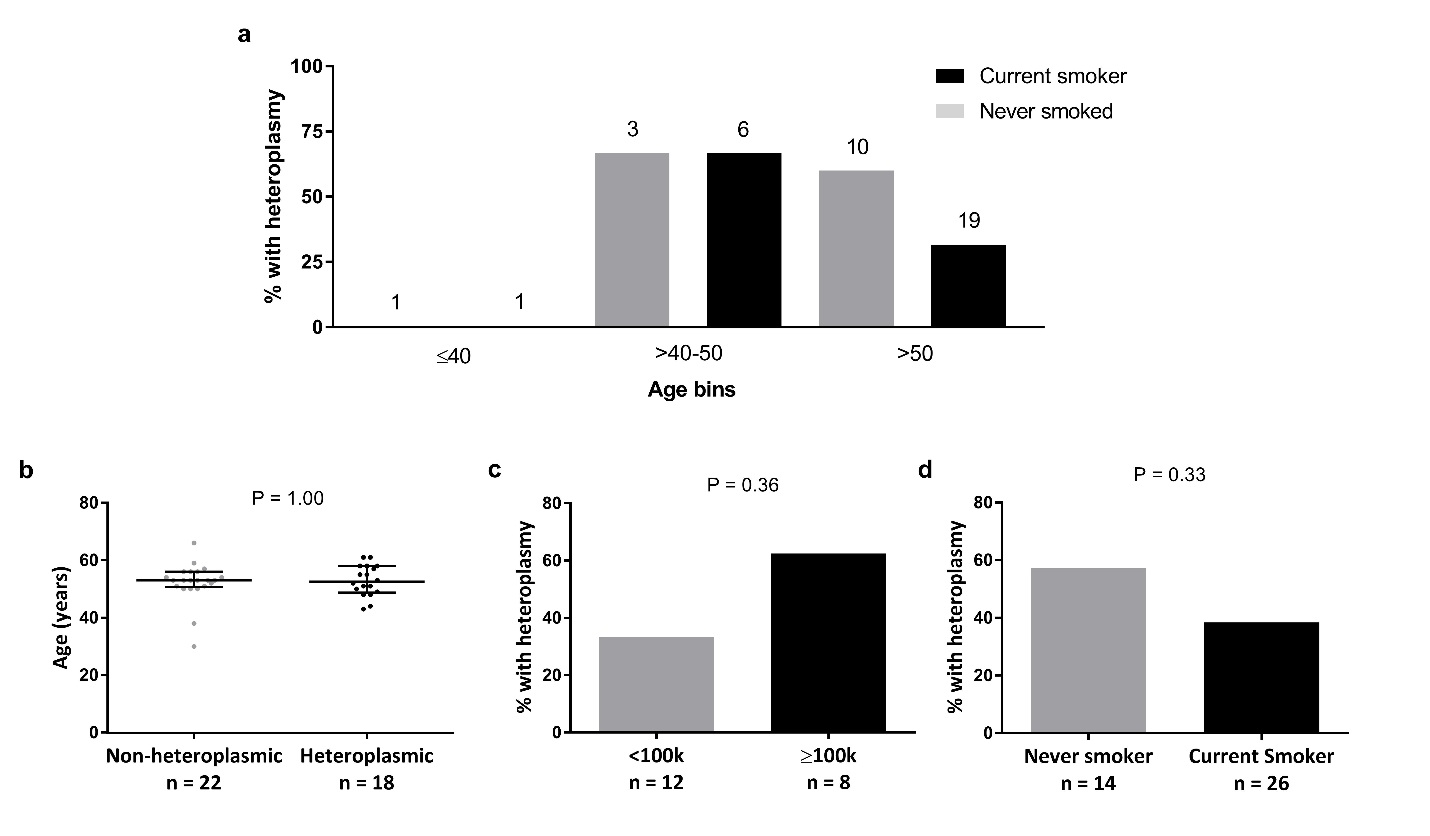


**Figure S4** Univariate tests in the Yale New Haven Hospital cohort alone, show no association between heteroplasmy, age, smoking, or HIV peak viral load. Heteroplasmy was categorized as a yes/no variable. To determine if this observation is reproducible, our heteroplasmy analysis was repeated in a second independent cohort using data obtained from a similar study of individuals living with HIV or not (Li et al., 2017). (**a**) Percentage of study participants with heteroplasmy binned by age and smoking status, with total number of participants for each bin (above bar), and percentage of those participants (bar height). The occurrence of heteroplasmy was not associated with age (**b**), HIV peak pVL (**c**), or smoking status (**d**). Mann-Whitney U tests and Fisher’s Exact tests were used in (b), (c) and (d) as appropriate.


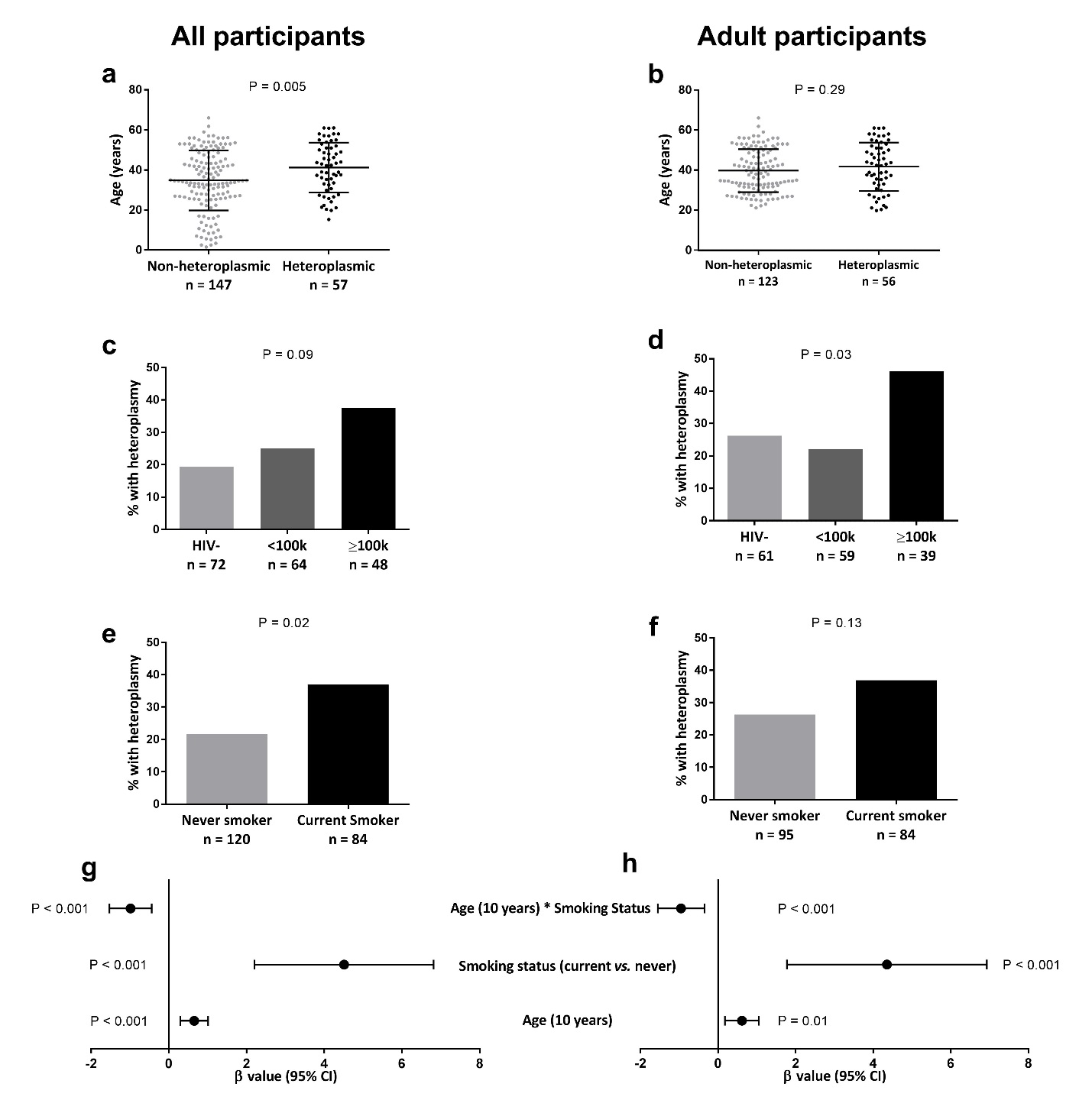


**Figure S5** “CARMA & Yale New Haven Hospital” cohort replicates the results observed in the CARMA cohort, with older chronological age and tobacco smoking associated with increased mtDNA heteroplasmy. Heteroplasmy was categorized as a yes/no variable. (**a, b**) The occurrence of heteroplasmy is univariatly associated with chronological age in all participants, but not in adults. (**c, d**) An association is observed between HIV and the occurrence of heteroplasmy among adult participants, however not among all participants. (**e, f**) Among all participants, current smokers show an increased occurrence of heteroplamsy (**e**) (P = 0.02), however this difference is not seen among adult participants (P = 0.13) (**f**). (**g, h**) Forest plot showing the estimated size of the effect (β value) and the 95% confidence interval on that estimate, based on an analysis of covariance. These models suggests that older age (P < 0.001), and smoking (P < 0.001) are associated with the occurrence of heteroplasmy (**g, h**). Furthermore, a significant interaction between smoking and age is observed in both models. These findings replicate the findings in the CARMA Cohort. Trichotomized HIV status was not added to any model, due to peak HIV viral load not being available for half of the 40 participants in the Yale New Haven Hospital cohort. Mann-Whitney U tests and Fisher’s Exact tests were used in (a), (b) (c), (d), (e) and (f) as appropriate.


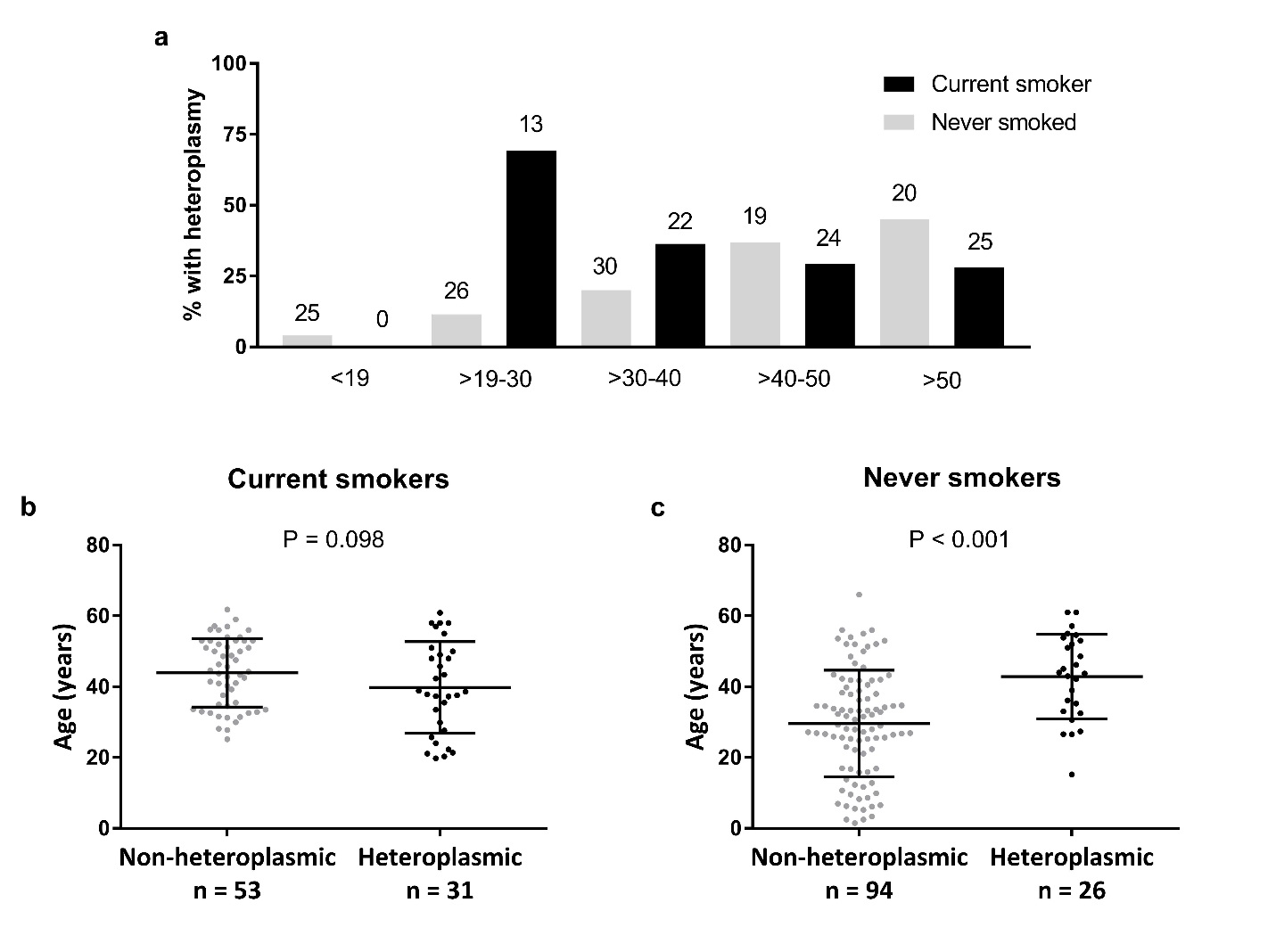


**Figure S6** The combined “CARMA & Yale New Haven Hospital” cohort shows a similar interaction between age and smoking, where current tobacco smokers experience a decrease in heteroplasmy with older age, while never smokers show the opposite. Heteroplasmy was categorized as a yes/no variable. (**a**) Percentage of study participants with heteroplasmy binned by age and smoking status, with total number of participants for each bin (above bar), and percentage of those participants (bar height). (**b, c**) Two sample t-tests among current smokers and never smokers to further understand the effect of age on heteroplasmy. Older age is associated with an increase in the occurrence of heteroplasmy (P < 0.001) among never smokers (**c**), while the reverse is seen as a non-significant trend for current smokers (P = 0.098) (**b**). This suggests an interaction whereby the effect of age on heteroplasmy is modulated by smoking status, supporting our finding in the CARMA cohort alone.

16535 gcccac acgttcccct taaataagac atcacga**T**g

1 gatcacaggt ctat**C**accc**T** atta**A**ccact cacgggagct ctccatgcat ttggtatttt

61 cgtCtggg**G**g gtAtg**C**acgc **G**atagcattg **C**gagacg**C**tg gaG**C**cggagc accctatgtc

121 gcagtatctg tctttgattc ct**GC**cTca**T**c cTAttattta tcgcacctac gttcaatatt

181 aCAggcgaAc atacTtacta aagTgtGtta attaattaat gc**T**tgtaGga catAataata

241 acaattgaat gtctg**C**acag **C**cActttcca cacagacatc ataacaaaaa atttccacca

301 aaccccccct cccccgcttc tggccacagc acttaaacac atctctgcca aaccccaaaa

361 acaaagaacc ctaacaccag cctaaccaga tttcaaattt tatcttttgg cggtatgcac

421 ttttaacagt caccccccaa ctaacacatt attttcccct cccactccca t

**Figure S7** Position of somatic mutations within clones (all, n=16) and participants (16 most commonly observed). Segment of the D-loop in the revised Cambridge reference sequence of the human mitochondrial genome (base pair 16560 to 279 inclusively, black font) analyzed in this study. Mitochondrial DNA sequence outside the region analyzed but within our amplicon is represented in orange. Positions showing a somatic mutation are capitalized and bolded if seen in the clones, underlined if seen in the participants. One mutation is seen in both. None of the 16 most mutated positions among study participants were within or adjacent to a homopolymer. However, 3 of the 16 positions mutated in the clones were so.


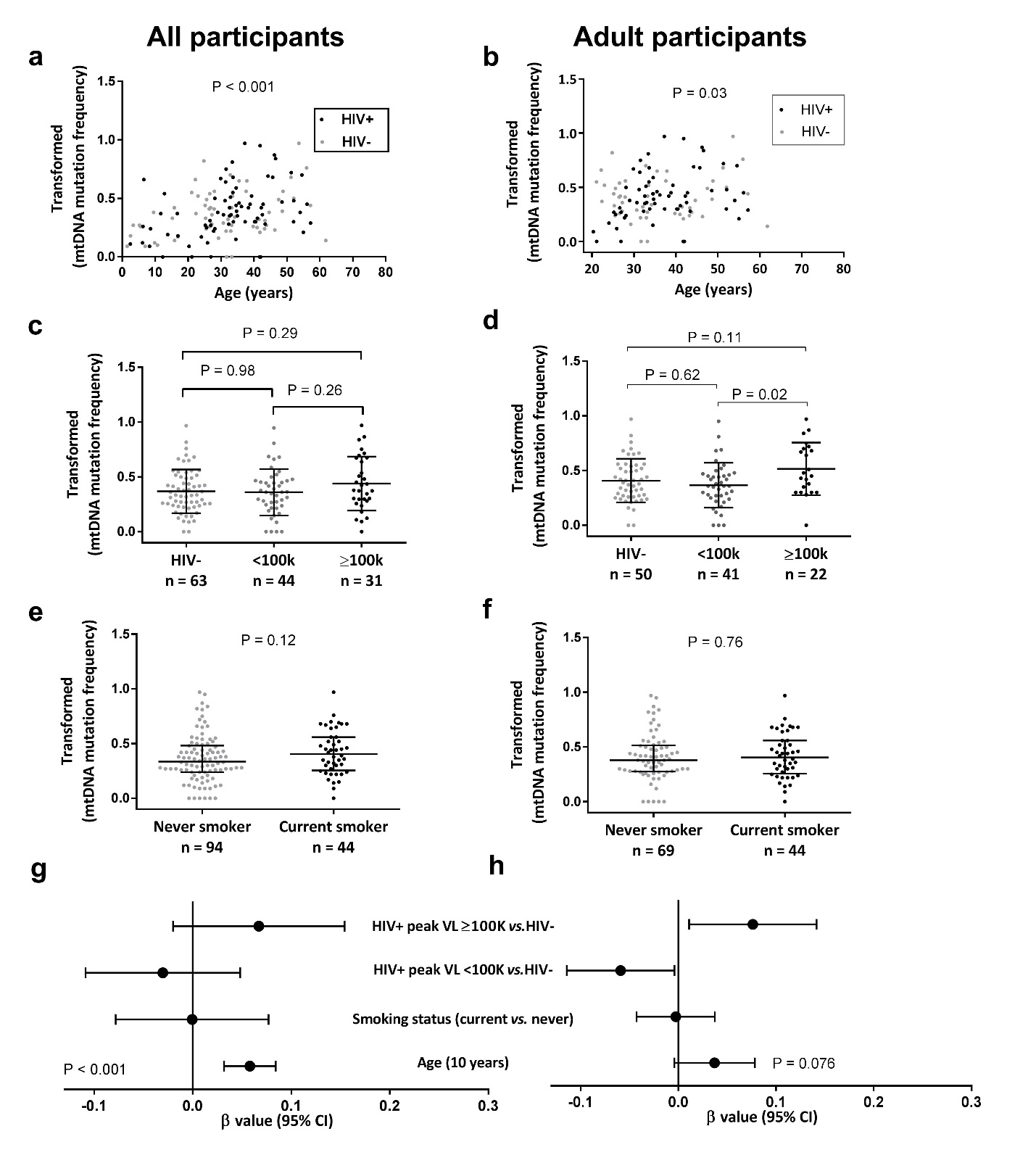


**Figure S8** Sensitivity analysis with the high stringency somatic mutation filter shows similar results. The measured frequencies (expressed as mutations per 10,000bp) were transformed (ln [x+1]) to normalize the distribution. (**a, b**) Blood somatic mtDNA substitutions are positively correlated with chronological age among all participants (**a**) and adult participants (**b**). (**c, d**) Only adult participants with a peak HIV plasma viral load ≥100,000 copies/mL showed a marginally higher somatic mtDNA substitution frequency compared to HIV-positive participants with a peak pVL <100,000 copies/mL (P = 0.02) or HIV-negative participants (P = 0.11), suggesting that having experienced high viremia may affect mtDNA mutations more so than living with the virus. (**e, f**) Tobacco smoking showed no association with somatic mtDNA mutation substitutions, as no difference was observed between participants who never smoked and those who were current smokers at the time of blood collection. (**g, h**) Forest plot showing the estimated size of the effect (β value) and the 95% confidence interval on that estimate, based on an least squares regression (**h**) and analysis of covariance (**g**). The models suggests that older age remains independently associated with increased somatic mtDNA mutations, even after adjusting for HIV and smoking in all participants (*P* < 0.001) but not in adult participants (*P* = 0.076). Among adults, having a peak pVL ≥ and <100,000 copies/mL remain associated with a higher and lower mtDNA mutation frequency, respectively. Smoking shows no association.

**TableS1** Univariate associations with transformed (ln [x+1]) blood somatic mtDNA substitution mutation frequency.

|  | **Study participants** | | | | | | | | |
| --- | --- | --- | --- | --- | --- | --- | --- | --- | --- |
|  | **HIV+ and HIV-** | | | | | | **HIV+ only** | | |
|  | **All participants**  **(*n* = 164)** | | | **All Adult (≥19y)**  **(*n* = 139)** | | | **Adult (≥19y)**  **(*n* = 80)** | | |
| **Parameters** | **model** | **Transformation** | ***p*-value** | **model** | **Transformation** | ***p*-value** | **model** | **Transformation** | ***p*-value** |
| **Age** (years) | Linear Regression | None | <0.001 | Linear Regression | None | 0.001 | Linear Regression | None | 0.015 |
| **BMI**(kg/m^2^) | Linear Regression | LOG_e_ | 0.620 | Linear Regression | LOG_e_ | 0.620 | Linear Regression | LOG_e_ | 0.926 |
| **HIV status** (HIV+ *vs.* HIV-) | Two Sample t-test | None | 0.153 | Two Sample t-test | None | 0.225 |  |  |  |
| **HIV status** (HIV- vs. HIV+ <100k vs. HIV+ ≥100k) | One-way ANOVA | None | 0.143 | One-way ANOVA | None | 0.039 |  |  |  |
| **Smoking status** (current vs. Never) | Mann-Whitney test | None | 0.244 | Two Sample t-test | None | 0.777 | Two sample t-test | None | 0.849 |
| **Alcohol use** (drink-year) | Spearman Correlation | None | 0.005 | Spearman Correlation | None | 0.431 | Spearman Correlation | None | 0.084 |
| **Drug use** (weekly/daily) | Mann-Whitney test | None | 0.084 | Two Sample t-test | None | 0.447 | Mann-Whitney test | None | 0.945 |
| **Ethnicity*** | Kruskal-Wallis test | None | 0.247 | Kruskal-Wallis test | None | 0.461 | Kruskal-Wallis test | None | 0.592 |
| **Current CD4 cell count** (cells/µl) |  |  |  |  |  |  | Linear Regression | None | 0.320 |
| **CD4 nadir** (cells/µl) |  |  |  |  |  |  | Spearman Regression | None | 0.074 |
| **Current HIV pVL** (≥50 vs <50 copies/ml) |  |  |  |  |  |  | Two sample t-test | None | 0.481 |
| **Peak HIV pVL**  (<100k vs ≥100k copies/ml) |  |  |  |  |  |  | Two sample t-test | None | 0.035 |
| **HIV treatment status** (PI vs NNRTI vs off) |  |  |  |  |  |  | One-way ANOVA | None | 0.853 |

*Participants with either unknown or other ethnicity were excluded from analysis. Ethnicity was not examined among children as ethnicity data was missing for all anonymous controls. Ethnicity was categorized as Caucasian, African/Caribbean/Black, Indigenous, Asian, or unknown; Combination antiretroviral therapy (cART) was categorized as protease inhibitor (PI)-based, non-nucleoside reverse transcriptase inhibitor (NNRTI)-based, or not currently on cART (off); Peak HIV plasma viral load (pVL).

**Table S2** Demographic information for study participants showing mtDNA heteroplasmic substitution mutations in the D-loop according to the revised Cambridge reference sequence.

| **Sample ID** | **Position (mutation)** | ***n** (%)** | **Age (years)** | **HIV**  **status** | **Smoking status** | **Current CD4 count (cells/µl)** | **CD4 nadir (cells/µl)** | **Current HIV pVL** | **Peak HIV pVL** | **HIV treatment status** |
| --- | --- | --- | --- | --- | --- | --- | --- | --- | --- | --- |
| **J-1-16** | 146 (t to c) | 9 (2) | 57 | HIV+ | Never | 680 | 230 | 39 | <100 000 | NNRTI |
| **J-2-15** | 151 (t to c) | 89 (23) | 24 | HIV+ | Current | 890 | 490 | 347 | <100 000 | Off ART |
| **J-3-16** | 153 (a to g) | 9 (2) | 37 | HIV+ | Current | 300 | 70 | 24288 | ≥100 000 | Off ART |
| **J-3-3** | 214 (a to g) | 30 (9) | 39 | HIV+ | Current | 300 | 90 | 39 | ≥100 000 | PI |
| **J-8-14** | 240 (a to g) | 10 (3) | 15 | HIV- | Never |  |  |  |  |  |
| **J-8-5** | 150 (c to t) | 41 (11) | 30 | HIV+ | Current | 550 | 190 | 39 | ≥100 000 | PI |
| **M-2-13** | 146 (t to c) | 59 (18) | 38 | HIV- | Current |  |  |  |  |  |
| **M-2-14** | 200 (g to a) | 10 (2) | 37 | HIV+ | Current | 210 | 200 | 611 | ≥100 000 | Off ART |
| **M-2-15** | 73 (a to g) | 15 (7) | 21 | HIV+ | Current | 280 | 170 | 2287 | <100 000 | Off ART |
| **M-2-16** | 72 (c to t)  146 (t to c)  185 (a to g) | 8 (3)  9 (4)  9 (4) | 27 | HIV- | Never |  |  |  |  |  |
| **M-2-3** | 64 (c to t)  153 (a to g)  207 (a to g)  235 (a to g) | 26 (11)  26 (11)  26 (11)  25 (11) | 39 | HIV+ | Never | 600 | 280 | 39 | <100 000 | NNRTI |
| **M-2-4** | 146 (c to t) | 16 (5) | 31 | HIV- | Never |  |  |  |  |  |
| **M-2-6** | 64 (c to t)  146 (t to c)  153 (a to g) | 11 (3)  11 (3)  11 (3) | 46 | HIV+ | Never | 510 | 260 | 39 | ≥100 000 | PI |
| **M-2-7** | 64 (t to c)  73 (a to g)  95 (g to a)  150.1 (c to t)  151 (c to t)  182 (c to t)  195 (t to c)  207 (a to g)  235 (a to g)  236 (c to t) | 127 (33)  127 (33)  127 (33)  106 (27)  20 (5)  127 (33)  127 (33)  127 (33)  127 (33)  127 (33) | 33 | HIV+ | Never | 330 | 160 | 600 | <100 000 | PI |
| **M-3-10** | 64 (c to t)  146 (t to c)  195 (c to t)  200 (g to a)  225 (a to g)  235 (a to g) | 7 (2)  8 (3)  7 (2)  10 (3)  8 (3)  7 (2) | 36 | HIV- | Current |  |  |  |  |  |
| **M-4-10** | 72 (c to t)  152 (t to c) | 107 (27)  107 (27) | 27 | HIV- | Never |  |  |  |  |  |
| **M-4-13** | 73 (a to g)  146 (c to t)  182 (c to t)  195 (t to c)  198 (c to t)  204 (t to c) | 144 (40)  143 (39)  144 (40)  143 (39)  143 (39)  143 (39) | 46 | HIV+ | Current | 590 | 470 | 1164 | <100 000 | Off ART |
| **M-4-14** | 207 (g to a) | 115 (33) | 43 | HIV+ | Current | 720 | 100 | 202 | ≥100 000 | PI |
| **M-4-15** | 72 (c to t)  73 (a to g)  200 (g to a)  228 (g to a) | 134 (37)  134 (37)  136 (37)  134 (37) | 38 | HIV- | Current |  |  |  |  |  |
| **M-4-16** | 146 (c to t)  185 (g to a)  189 (g to a)  228 (g to a) | 116 (28)  116 (28)  117 (28)  116 (28) | 28 | HIV+ | Current | 530 | 490 | 14179 | <100 000 | Off ART |
| **M-4-17** | 146 (t to c)  189 (a to g) | 204 (36)  203 (36) | 36 | HIV+ | Never | 510 | 170 | 39 | <100 000 | PI |
| **M-4-3** | 73 (a to g)  239 (c to t) | 179 (47)  179 (47) | 26 | HIV+ | Current | 690 | 200 | 39 | ≥100 000 | PI |
| **M-4-4** | 152 (c to t) | 218 (53) | 35 | HIV- | Never |  |  |  |  |  |
| **M-4-5** | 114 (c to t) | 68 (14) | 21 | HIV- | Current |  |  |  |  |  |
| **M-4-6** | 204 (t to c) | 170 (44) | 20 | HIV+ | Current | 810 | 610 | 39 | <100 000 | Off ART |
| **M-4-7** | 73 (a to g)  204 (c to t)  239 (c to t) | 232 (50)  232 (50)  231 (50) | 45 | HIV- | Never |  |  |  |  |  |
| **M-5-17** | 152 (t to c) | 10 (3) | 33 | HIV+ | Never | 450 | 191 | 39 | <100 000 | PI |
| **M-6-10** | 146 (t to c) | 10 (3) | 55 | HIV+ | Never |  | 260 |  | ≥100 000 | NNRTI |
| **M-7-10** | 72 (c to t)  152 (t to c) | 178 (44)  179 (44) | 61 | HIV+ | Current | 380 | 80 | 39 | <100 000 | NNRTI |
| **M-7-13** | 73 (g to a)  146 (t to c)  182 (t to c)  195 (c to t)  198 (t to c)  204 (c to t) | 165 (37)  174 (39)  172 (39)  173 (39)  173 (39)  172 (39) | 42 | HIV+ | Never | 120 | 40 | 33916 | ≥100 000 | Off ART |
| **M-7-14** | 207 (a to g) | 172 (36) | 44 | HIV+ | Never | 700 | 20 | 39 | ≥100 000 | PI |
| **M-7-15** | 70.1 (t to c)  72 (t to c)  73 (g to a)  200 (a to g)  228 (a to g) | 34 (7)  152 (32)  153 (32)  190 (40)  191 (40) | 42 | HIV+ | Current | 140 | 50 | 39 | ≥100 000 | PI |
| **M-7-16** | 146 (t to c)  185 (a to g)  189 (a to g)  228 (a to g) | 242 (45)  243 (46)  241 (45)  242 (45) | 39 | HIV- | Current |  |  |  |  |  |
| **M-7-17** | 146 (t to c)  189 (a to g) | 317 (52)  317 (52) | 22 | HIV+ | Current | 160 | 120 | 16531 | <100 000 | PI |
| **M-7-3** | 73 (a to g)  239 (c to t) | 166 (36)  166 (36) | 54 | HIV+ | Never | 710 | 260 | 39 | ≥100 000 | NNRTI |
| **M-7-4** | 146 (c to t)  152 (c to t)  195 (t to c) | 205 (45)  197 (43)  205 (45) | 49 | HIV- | Never |  |  |  |  |  |
| **M-7-5** | 114 (t to c) | 296 (43) | 20 | HIV+ | Current | 800 | 400 | 425 | ≥100 000 | PI |
| **M-7-6** | 204 (t to c) | 141 (30) | 27 | HIV- | Never |  |  |  |  |  |
| **M-7-7** | 73 (a to g)  204 (c to t)  239 (c to t) | 199 (36)  199 (36)  198 (36) | 33 | HIV- | Current |  |  |  |  |  |

*Number of PID consensus sequences containing a given mutation.

**Table S3** Univariate associations with blood heteroplasmic mtDNA substitution mutation frequency.

|  | **Study participants** | | | | | | | | |
| --- | --- | --- | --- | --- | --- | --- | --- | --- | --- |
|  | **HIV+ and HIV-** | | | | | | **HIV+ only** | | |
|  | **All participants**  (*n* = 164) | | | **All Adult (≥19y)**  (*n* = 139) | | | **Adult (≥19y)**  (*n* = 80) | | |
| **Parameters** | **Model** | **Transformation** | **P value** | **Model** | **Transformation** | **P value** | **Model** | **Transformation** | **P value** |
| **Age (years)** | Mann-Whitney | None | 0.180 | Mann-Whitney | None | 0.761 | Mann-Whitney | None | 0.992 |
| **BMI (kg/m^2^)** | Mann-Whitney | None | 0.281 | Mann-Whitney | None | 0.281 | Mann-Whitney | None | 0.756 |
| **HIV status (HIV+ *vs.* HIV-)** | Fisher’s Exact test | None | 0.273 | Fisher’s Exact test | None | 0.253 |  |  |  |
| **HIV status** (HIV- vs. HIV+ <100k vs.HIV+ ≥100k) | Fisher’s Exact test | None | 0.292 | Fisher’s Exact test | None | 0.128 |  |  |  |
| **Smoking status** (current vs. never) | Fisher’s Exact test | None | 0.007 | Fisher’s Exact test | None | 0.055 | Fisher’s Exact test | None | 0.028 |
| **Alcohol use** (drink-year) | Mann-Whitney | None | 0.072 | Mann-Whitney | None | 0.503 | Mann-Whitney | None | 0.113 |
| **Drug use** (weekly/daily) | Fisher’s Exact test | None | 0.229 | Fisher’s Exact test | None | 0.555 | Fisher’s Exact test | None | 0.067 |
| **Ethnicity*** | Fisher’s Exact test | None | 0.106 | Fisher’s Exact test | None | 0.239 | Fisher’s Exact test | None | 0.083 |
| **Current CD4 cell count** (cells/µl) |  |  |  |  |  |  | Mann-Whitney | None | 0.685 |
| **CD4 nadir** (cells/µl) |  |  |  |  |  |  | Mann-Whitney | None | 0.452 |
| **Current HIV pVL** (≥50 vs <50 copies/ml) |  |  |  |  |  |  | Fisher’s Exact test | None | 0.617 |
| **Peak HIV pVL**  (<100k vs ≥100k copies/ml) |  |  |  |  |  |  | Fisher’s Exact test | None | 0.138 |
| **HIV treatment status** (PI vs NNRTI vs off) |  |  |  |  |  |  | Fisher’s Exact test | None | 1.000 |

*Participants with either unknown or other ethnicity (17 for all, 4 for adults) were excluded from the analysis. Ethnicity was not examined among children as ethnicity data was missing for all anonymous controls. Ethnicity was categorized as Caucasian, African/Caribbean/Black, Indigenous, Asian, or unknown. Combination antiretroviral therapy (cART) was categorized as protease inhibitor (PI)-based, non-nucleoside reverse transcriptase inhibitor (NNRTI)-based, or not currently on cART (off); Peak HIV plasma viral load (pVL).

**Table S4** Primer used in PCR and qPCR reactions.

| **Primer ID** | **Sequence (5’-3’)** | **Use** |
| --- | --- | --- |
| Short KSF | CTCGAGGTCGACGGTATCG | Forward primer for qPCR to quantify PID-labeled mtDNA templates |
| MT48R | ccccccagacgaaaataccaaatg | Reverse primer for qPCR to quantify PID-labeled mtDNA templates (binds bp 48-71 in the D-loop region) |
| LAKSF | CCATCTCATCCCTGCGTGTCTCCGACTCAGCCTCGAGGTCGACGGTATCG | Forward primer used during PCR to generate amplicons for unidirectional GS FLX sequencing |
| LBDLR | CCTATCCCCTGTGTGCCTTGGCAGTCTCAGAGTatgggagtgrgagggraaaa | Reverse primer used during PCR to generate amplicons for unidirectional GS FLX sequencing (binds bp 451-474 in the D-loop region) |
| MT325F | cacagcacttaaacacatctctgc | Forward primer for qPCR quantification of mtDNA (binds bp 325-348 in the D-loop region) |
| MT474R | agtatgggagtgrgagggraaaa | Reverse primer for qPCR quantification of mtDNA (binds bp 451-474 in the D-loop region) |

Lower case indicates the region of the primer complementary to the human mtDNA sequence. All position numbers are given based on the revised Cambridge reference sequence.

**Table S5** Primer used to extend and label the original mtDNA template with both multiplex ID and PID.

| **Primer ID** | **Sequence (5’-3’)** |
| --- | --- |
| EP1 | CCTCGAGGTCGACGGTATCG**ACGAGTGCGT**NNNNCANNNNGTNNNNNgcccacacgttccccttaaataaga |
| EP3 | CCTCGAGGTCGACGGTATCG**AGACGCACTC**NNNNCANNNNGTNNNNNgcccacacgttccccttaaataaga |
| EP4 | CCTCGAGGTCGACGGTATCG**AGCACTGTAG**NNNNCANNNNGTNNNNNgcccacacgttccccttaaataaga |
| EP5 | CCTCGAGGTCGACGGTATCG**ATCAGACACG**NNNNCANNNNGTNNNNNgcccacacgttccccttaaataaga |
| EP6 | CCTCGAGGTCGACGGTATCG**ATATCGCGAG**NNNNCANNNNGTNNNNNgcccacacgttccccttaaataaga |
| EP7 | CCTCGAGGTCGACGGTATCG**CGTGTCTCTA**NNNNCANNNNGTNNNNNgcccacacgttccccttaaataaga |
| EP10 | CCTCGAGGTCGACGGTATCG**TCTCTATGCG**NNNNCANNNNGTNNNNNgcccacacgttccccttaaataaga |
| EP13 | CCTCGAGGTCGACGGTATCG**CATAGTAGTG**NNNNCANNNNGTNNNNNgcccacacgttccccttaaataaga |
| EP14 | CCTCGAGGTCGACGGTATCG**CGAGAGATAC**NNNNCANNNNGTNNNNNgcccacacgttccccttaaataaga |
| EP15 | CCTCGAGGTCGACGGTATCG**ATACGACGTA**NNNNCANNNNGTNNNNNgcccacacgttccccttaaataaga |

Lower case indicates the region of the primer complementary to the human mtDNA D-loop sequence (bp 16535 to 16559). Underlined bases represent the degenerate primer sequence. Bolded bases represent the MID region and the plain upper case indicates the KS region. All position numbers are given based on the revised Cambridge reference sequence.

**Table S6 Demographic and clinical characteristics of the study groups for all ages and adults in the sensitivity analysis with the high stringency somatic mutation filter.**

|  | **All participants** | | | **Adult participants** | | |
| --- | --- | --- | --- | --- | --- | --- |
| **Parameters** | **HIV +**  **(*n* = 75)** | **HIV –**  **(*n* = 63)** | ***p*-value** | **HIV +**  **(*n* = 63)** | **HIV –**  **(*n* = 50)** | ***p*-value** |
| **Age** (years) | 34 [27-42]  (2-57) | 31 [23-41]  (1-62) | 0.180 | 35 [31-42]  (20-57) | 33 [27-43]  (21-62) | 0.263 |
| **BMI** (kg/m^2^) |  |  |  | 26 [22-29]  (17-46) | 22 [21-27]  (16-38) | 0.018 |
|  |  |  |  |  |  |  |
| **Smoking status** |  |  | 1.000 |  |  | 0.990 |
| Current smoker | 24 (32) | 20 (32) |  | 24 (38) | 20 (40) |  |
| Never smoker | 51 (68) | 43 (68) |  | 39 (62) | 30 (60) |  |
| **Alcohol use** (drink-year) | 0 [0-2]  (0-352) | 2 [0-16] (0-424) | < 0.001 | 0 [0-6]  (0-352) | 3 [1-36] (0-424) | < 0.001 |
| **Drug use** (weekly-daily) |  |  | 0.183 |  |  | 0.109 |
| Current or past | 17 (23) | 22 (35) |  | 17 (27) | 22 (44) |  |
| Never | 56 (75) | 41 (65) |  | 44 (70) | 28 (56) |  |
|  |  |  |  |  |  |  |
| **Ethnicity** |  |  | < 0.001 |  |  | < 0.001 |
| Caucasian | 25 (33) | 24 (38) |  | 23 (37) | 24 (48) |  |
| African/Caribbean  /Black | 34 (45) | 0 (0) |  | 25 (40) | 0 (0) |  |
| Indigenous | 8 (11) | 14 (22) |  | 7 (11) | 14 (28) |  |
| Asian | 7 (9) | 10 (16) |  | 7 (11) | 10 (20) |  |
| Unknown | 1 (1) | 15 (24) |  | 1 (2) | 2 (4) |  |
| **CD4 cell count** (cells/µl) |  |  |  |  |  |  |
| Current | 507  [340-720]  (30-2350) |  |  | 455  [323-630]  (30-1570) |  |  |
|  |  |  |  |  |  |  |
| Nadir | 230  [160-315]  (0-2350) |  |  | 230  [160-300]  (0-1110) |  |  |
| **HIV pVL** (copies/ml) |  |  |  |  |  |  |
| Current pVL (<50) | 49 (65) |  |  | 37 (59) |  |  |
| Peak pVL > 100,000 | 31 (41) |  |  | 22 (35) |  |  |
| **HIV treatment status** |  |  |  |  |  |  |
| PI-based | 36 (48) |  |  | 28 (44) |  |  |
| NNRTI-based | 15 (20) |  |  | 12 (19) |  |  |
| On other cART | 2 (3) |  |  | 1 (2) |  |  |
| cART-naïve | 7 (9) |  |  | 7 (11) |  |  |
| Off cART | 15 (20) |  |  | 15 (24) |  |  |
|  |  |  |  |  |  |  |
|  |  |  |  |  |  |  |

Data presented as median [IQR] (range) or n (%); cART, combination antiretroviral therapy; PI, protease inhibitor; NNRTI, non-nucleoside reverse transcriptase inhibitor; BMI, body mass index; pVL, HIV plasma viral load <50 copies/ml is “undetectable.” BMI was unknown for 5 adults, drug use was unknown for 2 adults, and current CD4 cell count was unknown for 1 adult. Mann-Whitney test was used for age, BMI, and alcohol use. Chi-square test was used for smoking status, drug use, and ethnicity.

**Table S7** Univariate associations with transformed (ln(x+1)) blood somatic mtDNA substitution mutation frequency in the sensitivity analysis with the high stringency somatic mutation filter.

|  | **Study participants** | | | | | | | | |
| --- | --- | --- | --- | --- | --- | --- | --- | --- | --- |
|  | **HIV+ and HIV-** | | | | | | **HIV+ only** | | |
|  | **All participants**  (*n* = 138) | | | **All Adult (≥19y)**  (*n* = 113) | | | **Adult (≥19y)**  (*n* = 63) | | |
| **Parameters** | **Model** | **Transformation** | ***p*-value** | **Model** | **Transformation** | ***p*-value** | **Model** | **Transformation** | ***p*-value** |
| **Age** (years) | Linear Regression | None | <0.001 | Linear Regression | LOG_e_ | 0.03 | Linear Regression | None | 0.02 |
| **BMI**(kg/m^2^) |  |  |  | Spearman Correlation | None | 0.82 | Linear Regression | LOG_e_ | 0.88 |
| **HIV status** (HIV+ *vs.* HIV-) | Two Sample t-test | None | 0.51 | Two Sample t-test | None | 0.80 |  |  |  |
| **HIV status** (HIV- vs. HIV+ <100k vs. HIV+ ≥100k) | One-way ANOVA | None | 0.23 | One-way ANOVA | None | 0.03 | - | - | - |
| **Smoking status** (current vs. never) | Wilcoxon Rank-Sum test | None | 0.12 | Wilcoxon Rank-Sum test | None | 0.76 | Two sample t-test | None | 0.92 |
| **Alcohol use** (drink-year) | Spearman Correlation | None | 0.009 | Spearman Correlation | None | 0.569 | Spearman Correlation | None | 0.362 |
| **Drug use** (weekly/daily) | Wilcoxon Rank-Sum Test | None | 0.034 | Wilcoxon Rank-Sum Test | None | 0.307 | Wilcoxon Rank-Sum Test | None | 0.520 |
| **Ethnicity*** |  |  |  | Kruskal-Wallis Test | None | 0.91 | Kruskal-Wallis Test | None | 0.98 |
| **Current CD4^+^ cell count** (cells/µl) |  |  |  |  |  |  | Linear Regression | LOG_e_ | 0.15 |
| **CD4 nadir** (cells/µl) |  |  |  |  |  |  | Linear Regression | Arrhenius | 0.08 |
| **Current HIV pVL** (≥50 vs <50 copies/ml) |  |  |  |  |  |  | Two sample t-test | None | 0.58 |
| **Peak HIV pVL**  (<100k vs ≥100k copies/ml) |  |  |  |  |  |  | Two sample t-test | None | 0.02 |
| **Current cART regimen** (PI vs NNRTI vs off) |  |  |  |  |  |  | One-way ANOVA | None | 0.66 |

*Participants with either unknown or other ethnicity were excluded from analysis. Ethnicity was not examined among children as ethnicity data was missing for all anonymous controls. Ethnicity was categorized as Caucasian, African/Caribbean/Black, Indigenous, Asian, or unknown; Combination antiretroviral therapy (cART) was categorized as protease inhibitor (PI)-based, non-nucleoside reverse transcriptase inhibitor (NNRTI)-based, or not currently on cART (off); peak HIV plasma viral load (pVL).

**Table S8** Demographic information for study participants showing mtDNA heteroplasmic substitution mutations in the sensitivity analysis with the high stringency somatic mutation filter.

| **Sample ID** | **Position (mutation)** | ***n**(%)** | **Age**  (years) | **HIV  status** | **Smoking status** | **Current CD4**  (cells/µl) | **CD4 nadir**  (cells/µl) | **Current HIV pVL**  (copies/ml) | **Peak HIV pVL**  (copies/ml) | **HIV treatment status** |
| --- | --- | --- | --- | --- | --- | --- | --- | --- | --- | --- |
| **J-8-5** | 150 (c to t) | 41 (11) | 30 | HIV+ | Current | 550 | 190 | 39 | ≥100 000 | PI |
| **M-5-17** | 152 (t to c) | 10 (3) | 33 | HIV+ | Never | 450 | 191 | 39 | <100 000 | PI |
| **M-6-10** | 146 (t to c) | 10 (3) | 55 | HIV+ | Never |  | 260 |  | ≥100 000 | NNRTI |
| **J-2-15** | 151 (t to c) | 89 (23) | 24 | HIV+ | Current | 890 | 490 | 347 | <100 000 | Off ART |
| **J-8-14** | 240 (a to g) | 10 (3) | 15 | HIV- | Never |  |  |  |  |  |
| **J-3-16** | 153 (a to g) | 9 (2) | 37 | HIV+ | Current | 300 | 70 | 24288 | ≥100 000 | Off ART |
| **M-4-6** | 204 (t to c) | 170 (44) | 20 | HIV+ | Current | 810 | 610 | 39 | <100 000 | Off ART |
| **M-7-6** | 204 (t to c) | 141 (30) | 27 | HIV- | Never |  |  |  |  |  |
| **M-2-15** | 73 (a to g) | 15 (7) | 21 | HIV+ | Current | 280 | 170 | 2287 | <100 000 | Off ART |
| **M-2-4** | 146 (c to t) | 16 (5) | 31 | HIV- | Never |  |  |  |  |  |

*Number of PID consensus sequences containing a given mutation.

**Table S9** Contamination information for study participants in the sensitivity analysis with the high stringency somatic mutation filter.

| **Sample ID** | **PID consensus*** | **PID consensus^†^** | **Inclusion status** | **Age** (years) | **HIV status** | **Smoking status** | **Current CD4 count** (cells/µl) | **CD4 nadir** (cells/µl) | **Current HIV pVL** (copies/ml) | **Peak HIV pVL** (copies/ml) | **HIV treatment status** |
| --- | --- | --- | --- | --- | --- | --- | --- | --- | --- | --- | --- |
| **M-8-13** | 0 | 0 | Included | 16 | HIV- | Never |  |  |  |  |  |
| **J-4-5** | 0 | 0 | Included | 5 | HIV- | Never |  |  |  |  |  |
| **M-8-10** | 1 | 1 | Included | 1 | HIV- | Never |  |  |  |  |  |
| **J-3-15** | 0 | 0 | Included | 11 | HIV- | Never |  |  |  |  |  |
| **J-6-3** | 1 | 0 | Included | 3 | HIV- | Never |  |  |  |  |  |
| **J-7-7** | 0 | 0 | Included | 2 | HIV- | Never |  |  |  |  |  |
| **J-8-4** | 0 | 0 | Included | 16 | HIV- | Never |  |  |  |  |  |
| **J-8-14** | 0 | 0 | Included | 15 | HIV- | Never |  |  |  |  |  |
| **J-2-14** | 1 | 1 | Included | 6 | HIV- | Never |  |  |  |  |  |
| **J-4-14** | 1 | 0 | Included | 7 | HIV- | Never |  |  |  |  |  |
| **M-3-4** | 1 | 1 | Included | 10 | HIV- | Never |  |  |  |  |  |
| **J-8-3** | 0 | 0 | Included | 17 | HIV- | Never |  |  |  |  |  |
| **J-5-10** | 0 | 0 | Included | 10 | HIV- | Never |  |  |  |  |  |
| **J-3-3** | 28 | 0 | Excluded | 39 | HIV+ | Current | 300 | 90 | Undetectable | ≥100 000 | PI |
| **M-7-17** | 313 | 313 | Excluded | 22 | HIV+ | Current | 160 | 120 | Detectable | <100 000 | PI |
| **M-2-15** | 15 | 0 | Included | 21 | HIV+ | Current | 280 | 170 | Detectable | <100 000 | Off ART |
| **M-8-6** | 0 | 0 | Included | 51 | HIV+ | Never | 790 | 0 | Undetectable | ≥100 000 | PI |
| **J-1-17** | 2 | 2 | Excluded | 45 | HIV+ | Never | 350 | 250 | Undetectable | <100 000 | NNRTI |
| **M-2-17** | 3 | 2 | Included | 39 | HIV+ | Never | 720 | 170 | Undetectable | <100 000 | PI |
| **J-2-15** | 0 | 0 | Included | 24 | HIV+ | Current | 890 | 490 | Detectable | <100 000 | Off ART |
| **J-2-7** | 0 | 0 | Included | 28 | HIV+ | Current | 460 | 252 |  | <100 000 | PI |
| **M-7-5** | 292 | 0 | Excluded | 20 | HIV+ | Current | 800 | 400 | Detectable | ≥100 000 | PI |
| **M-5-10** | 0 | 0 | Included | 41 | HIV+ | Current | 540 | 280 | Undetectable | <100 000 | PI |
| **M-7-15** | 173 | 173 | Excluded | 42 | HIV+ | Current | 140 | 50 | Undetectable | ≥100 000 | PI |
| **J-5-14** | 2 | 0 | Included | 46 | HIV+ | Current | 160 | 90 | Undetectable | ≥100 000 | PI |
| **M-4-6** | 0 | 0 | Included | 20 | HIV+ | Current | 810 | 610 | Undetectable | <100 000 | Off ART |
| **J-2-13** | 1 | 0 | Included | 34 | HIV+ | Never | 500 | 120 | Undetectable | <100 000 | PI |
| **J-7-16** | 2 | 0 | Included | 43 | HIV+ | Current | 400 | 320 | Detectable | ≥100 000 | Off ART |
| **M-3-7** | 0 | 0 | Included | 42 | HIV+ | Never | 460 | 180 | Undetectable | ≥100 000 | NNRTI |
| **M-8-7** | 0 | 0 | Included | 34 | HIV+ | Never | 1120 | 731 | Undetectable | ≥100 000 | NNRTI |
| **M-6-6** | 0 | 0 | Included | 33 | HIV+ | Never | 340 | 160 | Undetectable | ≥100 000 | PI |
| **M-2-6** | 11 | 10 | Included | 46 | HIV+ | Never | 510 | 260 | Undetectable | ≥100 000 | PI |
| **M-5-3** | 3 | 2 | Included | 35 | HIV+ | Current | 300 | 150 | Detectable | ≥100 000 | PI |
| **J-4-7** | 0 | 0 | Included | 31 | HIV+ | Never | 580 | 310 | Detectable | <100 000 | PI |
| **J-3-4** | 1 | 1 | Included | 40 | HIV+ | Current | 340 | 160 | Undetectable | ≥100 000 | PI |
| **M-4-3** | 179 | 178 | Excluded | 26 | HIV+ | Current | 690 | 200 | Undetectable | ≥100 000 | PI |
| **M-1-17** | 1 | 0 | Included | 25 | HIV+ | Never | 800 | 220 | Undetectable | <100 000 | PI |
| **M-5-13** | 0 | 0 | Included | 27 | HIV+ | Never | 470 | 440 | Detectable | <100 000 | Off ART |
| **J-6-14** | 1 | 0 | Included | 29 | HIV+ | Never | 390 | 240 | Undetectable | <100 000 | PI |
| **M-5-7** | 1 | 0 | Included | 40 | HIV+ | Never | 400 | 230 | Detectable | ≥100 000 | PI |
| **J-6-10** | 1 | 0 | Included | 35 | HIV+ | Never | 440 | 180 | Detectable | <100 000 | Off ART |
| **M-6-14** | 2 | 0 | Included | 33 | HIV+ | Never | 1160 | 662 | Detectable | <100 000 | Off ART |
| **J-6-6** | 1 | 0 | Included | 35 | HIV+ | Never | 290 | 150 | Undetectable | <100 000 | PI |
| **M-4-17** | 200 | 198 | Excluded | 36 | HIV+ | Never | 510 | 170 | Undetectable | <100 000 | PI |
| **J-8-5** | 0 | 0 | Included | 30 | HIV+ | Current | 550 | 190 | Undetectable | ≥100 000 | PI |
| **M-8-15** | 0 | 0 | Included | 32 | HIV+ | Never | 270 | 240 | Detectable | ≥100 000 | Off ART |
| **M-7-13** | 169 | 169 | Excluded | 42 | HIV+ | Never | 120 | 40 | Detectable | ≥100 000 | Off ART |
| **J-3-16** | 1 | 0 | Included | 37 | HIV+ | Current | 300 | 70 | Detectable | ≥100 000 | Off ART |
| **M-7-14** | 167 | 1 | Excluded | 44 | HIV+ | Never | 700 | 20 | Undetectable | ≥100 000 | PI |
| **J-1-16** | 9 | 8 | Included | 57 | HIV+ | Never | 680 | 230 | Undetectable | <100 000 | NNRTI |
| **M-2-14** | 6 | 6 | Included | 37 | HIV+ | Current | 210 | 200 | Detectable | ≥100 000 | Off ART |
| **J-8-10** | 2 | 0 | Included | 34 | HIV+ | Never | 510 | 300 | Undetectable | <100 000 | PI |
| **M-8-14** | 6 | 4 | Included | 38 | HIV+ | Never | 620 | 230 | Undetectable | ≥100 000 | NNRTI |
| **J-8-16** | 0 | 0 | Included | 12 | HIV+ | Never | 720 | 250 | Undetectable | <100 000 | NNRTI |
| **M-7-3** | 167 | 165 | Excluded | 54 | HIV+ | Never | 710 | 260 | Undetectable | ≥100 000 | NNRTI |
| **J-2-17** | 1 | 0 | Included | 17 | HIV+ | Never | 860 | 120 | Undetectable | ≥100 000 | PI |
| **M-5-14** | 1 | 1 | Included | 52 | HIV+ | Never | 340 | 30 | Undetectable | <100 000 | PI |
| **J-5-15** | 1 | 1 | Included | 26 | HIV+ | Never | 520 | 520 | Detectable | <100 000 | Off ART |
| **M-8-3** | 4 | 2 | Included | 28 | HIV+ | Never | 280 | 30 | Undetectable | <100 000 | PI |
| **M-1-4** | 5 | 2 | Included | 35 | HIV+ | Current | 430 | 200 | Undetectable | <100 000 | NNRTI |
| **M-4-14** | 115 | 1 | Excluded | 43 | HIV+ | Current | 720 | 100 | Detectable | ≥100 000 | PI |
| **M-3-5** | 2 | 0 | Included | 36 | HIV+ | Never | 750 | 49 | Undetectable | ≥100 000 | PI |
| **J-5-13** | 0 | 0 | Included | 28 | HIV+ | Current | 330 | 320 | Detectable | <100 000 | Off ART |
| **M-3-16** | 0 | 0 | Included | 42 | HIV+ | Never | 450 | 270 | Undetectable | <100 000 | NNRTI |
| **M-2-7** | 126 | 126 | Excluded | 33 | HIV+ | Never | 330 | 160 | Detectable | <100 000 | PI |
| **M-1-6** | 2 | 0 | Included | 39 | HIV+ | Current | 630 | 190 | Undetectable | <100 000 | PI |
| **J-7-14** | 1 | 1 | Included | 33 | HIV+ | Current | 810 | 550 | Detectable | <100 000 | Off ART |
| **M-7-10** | 175 | 174 | Excluded | 61 | HIV+ | Current | 380 | 80 | Undetectable | <100 000 | NNRTI |
| **M-5-17** | 1 | 0 | Included | 33 | HIV+ | Never | 450 | 191 | Undetectable | <100 000 | PI |
| **M-1-16** | 0 | 0 | Included | 42 | HIV+ | Never | 630 | 230 | Undetectable | <100 000 | NNRTI |
| **M-3-15** | 2 | 2 | Included | 35 | HIV+ | Current | 260 | 190 | Undetectable | <100 000 | PI |
| **M-6-4** | 2 | 2 | Included | 27 | HIV+ | Never | 440 | 440 | Undetectable | <100 000 | Off ART |
| **M-4-16** | 116 | 115 | Excluded | 28 | HIV+ | Current | 530 | 490 | Detectable | <100 000 | Off ART |
| **J-6-7** | 5 | 3 | Included | 31 | HIV+ | Current | 30 | 30 | Detectable | ≥100 000 | Off ART |
| **J-3-7** | 2 | 0 | Included | 32 | HIV+ | Never | 200 | 200 | Detectable | ≥100 000 | PI |
| **M-6-10** | 13 | 0 | Included | 55 | HIV+ | Never |  | 260 |  | ≥100 000 | NNRTI |
| **M-6-3** | 0 | 0 | Included | 43 | HIV+ | Never | 160 | 160 | Undetectable | <100 000 | NNRTI |
| **J-6-16** | 0 | 0 | Included | 54 | HIV+ | Never | 560 | 240 | Undetectable | ≥100 000 | PI |
| **M-4-13** | 144 | 143 | Excluded | 46 | HIV+ | Current | 590 | 470 | Detectable | <100 000 | Off ART |
| **J-8-6** | 4 | 0 | Included | 44 | HIV+ | Current | 300 | 300 | Detectable | <100 000 | Off ART |
| **J-3-6** | 3 | 0 | Included | 31 | HIV+ | Current | 700 | 77 | Detectable | ≥100 000 | PI |
| **J-8-15** | 4 | 1 | Included | 33 | HIV+ | Current | 220 | 80 | Undetectable | <100 000 | NNRTI |
| **J-3-17** | 2 | 0 | Included | 35 | HIV+ | Never | 1070 | 900 | Undetectable | <100 000 | Off ART |
| **M-8-4** | 0 | 0 | Included | 26 | HIV+ | Never | 690 | 260 | Undetectable | <100 000 | PI |
| **M-6-16** | 0 | 0 | Included | 42 | HIV+ | Never | 510 | 270 | Undetectable | <100 000 | NNRTI |
| **J-3-14** | 1 | 0 | Included | 27 | HIV+ | Never | 1570 | 1110 | Detectable | <100 000 | Off ART |
| **M-2-5** | 5 | 4 | Included | 56 | HIV+ | Current | 450 | 350 | Detectable | <100 000 | Off ART |
| **M-6-15** | 0 | 0 | Included | 32 | HIV- | Never |  |  |  |  |  |
| **J-4-16** | 1 | 0 | Included | 42 | HIV- | Never |  |  |  |  |  |
| **J-5-5** | 2 | 0 | Included | 25 | HIV- | Never |  |  |  |  |  |
| **M-8-5** | 2 | 0 | Included | 28 | HIV- | Never |  |  |  |  |  |
| **J-4-10** | 0 | 0 | Included | 43 | HIV- | Never |  |  |  |  |  |
| **J-7-6** | 0 | 0 | Included | 46 | HIV- | Current |  |  |  |  |  |
| **M-7-6** | 0 | 0 | Included | 27 | HIV- | Never |  |  |  |  |  |
| **M-5-16** | 0 | 0 | Included | 34 | HIV- | Never |  |  |  |  |  |
| **M-2-4** | 17 | 0 | Included | 31 | HIV- | Never |  |  |  |  |  |
| **M-7-4** | 193 | 193 | Excluded | 49 | HIV- | Never |  |  |  |  |  |
| **M-1-14** | 2 | 1 | Included | 32 | HIV- | Never |  |  |  |  |  |
| **J-4-6** | 1 | 0 | Included | 33 | HIV- | Never |  |  |  |  |  |
| **M-4-10** | 103 | 103 | Excluded | 27 | HIV- | Never |  |  |  |  |  |
| **J-6-17** | 1 | 1 | Included | 28 | HIV- | Never |  |  |  |  |  |
| **M-2-16** | 8 | 8 | Included | 27 | HIV- | Never |  |  |  |  |  |
| **J-2-10** | 1 | 0 | Included | 25 | HIV- | Never |  |  |  |  |  |
| **M-5-5** | 1 | 0 | Included | 25 | HIV- | Never |  |  |  |  |  |
| **J-7-10** | 0 | 0 | Included | 21 | HIV- | Never |  |  |  |  |  |
| **M-6-13** | 0 | 0 | Included | 27 | HIV- | Never |  |  |  |  |  |
| **M-6-17** | 0 | 0 | Included | 27 | HIV- | Never |  |  |  |  |  |
| **J-7-17** | 1 | 1 | Included | 26 | HIV- | Never |  |  |  |  |  |
| **J-4-17** | 0 | 0 | Included | 22 | HIV- | Never |  |  |  |  |  |
| **M-4-7** | 228 | 228 | Excluded | 45 | HIV- | Never |  |  |  |  |  |
| **M-6-5** | 1 | 0 | Included | 52 | HIV- | Never |  |  |  |  |  |
| **J-4-4** | 1 | 0 | Included | 27 | HIV- | Never |  |  |  |  |  |
| **J-3-13** | 1 | 0 | Included | 29 | HIV- | Never |  |  |  |  |  |
| **J-5-4** | 3 | 2 | Included | 33 | HIV- | Never |  |  |  |  |  |
| **M-6-7** | 1 | 0 | Included | 26 | HIV- | Never |  |  |  |  |  |
| **J-5-16** | 1 | 0 | Included | 38 | HIV- | Current |  |  |  |  |  |
| **M-3-14** | 0 | 0 | Included | 33 | HIV- | Current |  |  |  |  |  |
| **M-1-15** | 0 | 0 | Included | 33 | HIV- | Current |  |  |  |  |  |
| **J-7-13** | 0 | 0 | Included | 33 | HIV- | Current |  |  |  |  |  |
| **J-4-15** | 1 | 1 | Included | 43 | HIV- | Current |  |  |  |  |  |
| **J-4-13** | 2 | 0 | Included | 41 | HIV- | Current |  |  |  |  |  |
| **M-5-4** | 2 | 2 | Included | 33 | HIV- | Never |  |  |  |  |  |
| **J-4-3** | 1 | 1 | Included | 32 | HIV- | Current |  |  |  |  |  |
| **M-3-10** | 7 | 7 | Included | 36 | HIV- | Current |  |  |  |  |  |
| **M-7-7** | 196 | 196 | Excluded | 33 | HIV- | Current |  |  |  |  |  |
| **M-3-6** | 4 | 3 | Included | 48 | HIV- | Current |  |  |  |  |  |
| **M-7-16** | 240 | 239 | Excluded | 39 | HIV- | Current |  |  |  |  |  |
| **J-7-15** | 0 | 0 | Included | 44 | HIV- | Current |  |  |  |  |  |
| **J-7-4** | 1 | 0 | Included | 40 | HIV- | Never |  |  |  |  |  |
| **J-2-3** | 1 | 0 | Included | 22 | HIV- | Never |  |  |  |  |  |
| **J-8-13** | 0 | 0 | Included | 23 | HIV- | Never |  |  |  |  |  |
| **M-4-5** | 68 | 0 | Excluded | 21 | HIV- | Current |  |  |  |  |  |
| **J-5-7** | 0 | 0 | Included | 62 | HIV- | Current |  |  |  |  |  |
| **M-5-6** | 0 | 0 | Included | 25 | HIV- | Current |  |  |  |  |  |
| **M-4-4** | 214 | 0 | Excluded | 35 | HIV- | Never |  |  |  |  |  |
| **M-3-13** | 0 | 0 | Included | 38 | HIV- | Never |  |  |  |  |  |
| **M-2-10** | 11 | 5 | Included | 54 | HIV- | Never |  |  |  |  |  |
| **J-8-17** | 3 | 2 | Included | 51 | HIV- | Current |  |  |  |  |  |
| **J-2-5** | 0 | 0 | Included | 57 | HIV- | Current |  |  |  |  |  |
| **J-5-3** | 2 | 2 | Included | 56 | HIV- | Current |  |  |  |  |  |
| **J-1-13** | 1 | 0 | Included | 49 | HIV- | Current |  |  |  |  |  |
| **J-6-13** | 2 | 0 | Included | 45 | HIV- | Current |  |  |  |  |  |
| **J-5-17** | 0 | 0 | Included | 49 | HIV- | Current |  |  |  |  |  |
| **J-7-5** | 1 | 0 | Included | 41 | HIV- | Current |  |  |  |  |  |
| **M-4-15** | 136 | 134 | Excluded | 38 | HIV- | Current |  |  |  |  |  |
| **J-2-4** | 1 | 1 | Included | 2 | HIV+ | Never | 2350 | 2350 | Undetectable | ≥100 000 | PI |
| **M-8-16** | 0 | 0 | Included | 7 | HIV+ | Never | 255 | 144 | Undetectable | <100 000 | NNRTI |
| **J-6-15** | 0 | 0 | Included | 6 | HIV+ | Never | 1110 | 520 | Undetectable | ≥100 000 | PI |
| **J-6-5** | 0 | 0 | Included | 13 | HIV+ | Never | 504 | 216 | Undetectable | ≥100 000 | PI |
| **J-8-7** | 0 | 0 | Included | 6 | HIV+ | Never | 1080 | 1080 | Undetectable | ≥100 000 | PI |
| **J-5-6** | 0 | 0 | Included | 8 | HIV+ | Never | 1242 | 528 | Undetectable | ≥100 000 | PI |
| **J-3-5** | 0 | 0 | Included | 17 | HIV+ | Never | 1312 | 1 | Undetectable | ≥100 000 | Other |
| **J-2-16** | 1 | 1 | Included | 14 | HIV+ | Never | 682 | 252 | Undetectable | <100 000 | PI |
| **M-3-17** | 0 | 0 | Included | 12 | HIV+ | Never | 1060 | 100 | Undetectable | ≥100 000 | PI |
| **J-7-3** | 1 | 0 | Included | 9 | HIV+ | Never | 690 | 610 | Undetectable | ≥100 000 | NNRTI |
| **M-8-17** | 0 | 0 | Included | 52 | HIV+ | Never | 760 | 700 | Detectable | <100 000 | Off ART |
| **J-2-6** | 1 | 0 | Included | 43 | HIV+ | Current | 250 | 170 | Detectable | <100 000 | Off ART |
| **M-2-3** | 26 | 26 | Excluded | 39 | HIV+ | Never | 600 | 280 | Undetectable | <100 000 | NNRTI |
| **M-3-3** | 1 | 0 | Included | 34 | HIV+ | Current | 370 | 232 | Detectable | <100 000 | Off ART |
| **M-5-15** | 3 | 0 | Included | 47 | HIV+ | Never | 320 | 40 | Undetectable | ≥100 000 | Other |
| **J-3-10** | 1 | 0 | Included | 55 | HIV+ | Never | 550 | 250 | Undetectable | <100 000 | NNRTI |
| **J-6-4** | 2 | 1 | Included | 49 | HIV+ | Never | 380 | 110 | Undetectable | <100 000 | PI |
| **M-1-3** | 1 | 1 | Excluded | 37 | HIV+ | Never | 230 | 120 | Undetectable | ≥100 000 | PI |
| **M-2-13** | 58 | 2 | Excluded | 38 | HIV- | Current |  |  |  |  |  |

Number of PID consensus sequences containing *≥1 or ^†^ ≥2 mutations or and matching the sample consensus sequences of another sample.

**Table S10** Demographic characteristics of study participants enrolled in an independent cohort at Yale New Haven Hospital, New Haven, Connecticut, USA.

|  | **All participants (Paintsil)** | |
| --- | --- | --- |
| **Parameters** | **HIV +**  **(*n* = 47)** | **HIV –**  **(*n* = 25)** |
| **Age** (years) | 53 [50-57]  (30-72) | 51 [49-56]  (32-72) |
| **BMI** (kg/m^2^) | 27 [24-33] (17-83) |  |
|  |  |  |
| **Smoking status** |  |  |
| Current smoker | 26 (55) |  |
| Past smoker | 7 (15) |  |
| Never smoker | 14 (30) |  |
|  |  |  |
| **Ethnicity** |  |  |
| White nonhispanic | 13 (28) | 7 (28) |
| White hispanic | 4 (9) | 3 (12) |
| African american | 30 (64) | 15 (60) |
| **CD4^+^ cell count** (cells/µl) |  |  |
| Current | 616 [408-851] (86-1336) |  |
| Nadir | 260 [132-400] (10-1109) |  |
| **HIV plasma viral load** (copies/ml) |  |  |
| Current pvl (<50) | 38 (81) |  |
| Peak pVL >100,000 | 10 (38) |  |
| **HIV treatment status** |  |  |
| PI-based | 22 (47) |  |
| NNRTI-based | 18 (38) |  |
| On other cART | 7 (15) |  |
| cART-naive | 0 (0) |  |
| Off cART | 0 (0) |  |

Data presented as median [IQR] (range) or *n* (%); cART, combination antiretroviral therapy; PI, protease inhibitor; NNRTI, non‐nucleoside reverse transcriptase inhibitor; BMI, body mass index; pVL, HIV plasma viral load < 50 copies/ml is “undetectable.” BMI and smoking status were unknown for HIV-negative participants, CD4 nadir was unknown for 1 participant, HIV peak pVL was unknown for 21 participants

**REFFERENCES**

Brumme CJ, & Poon AFY (2017) Promises and pitfalls of Illumina sequencing for HIV resistance genotyping. *Virus Res* 239, 97–105.

Li, M., Foli, Y., Liu, Z., Wang, G., Hu, Y., Lu, Q., … Paintsil, E. (2017). High frequency of mitochondrial DNA mutations in HIV‐infected treatment‐experienced individuals. *HIV Medicine, 18*(1), 45–55. https://doi.org/10.1111/hiv.12390

Zanet, D. L., Thorne, A., Singer, J., Maan, E. J., Sattha, B., Le Campion, A., … Cote, H. C. F. (2014). Association between short leukocyte telomere length and HIV infection in a cohort study: No evidence of a relationship with antiretroviral therapy. *Clinical Infectious Diseases : An Official Publication of the Infectious Diseases Society of America*, *58*(9), 1322–1332. https://doi.org/10.1093/cid/ciu051
